# Valproate inhibits MAP kinase signalling and cell cycle progression in *S. cerevisiae*

**DOI:** 10.1038/srep36013

**Published:** 2016-10-26

**Authors:** Kristelle Desfossés-Baron, Ian Hammond-Martel, Antoine Simoneau, Adnane Sellam, Stephen Roberts, Hugo Wurtele

**Affiliations:** 1 Maisonneuve-Rosemont Hospital Research Center, 5415 L’Assomption Boulevard, Montreal, H1T 2M4, Canada; 2 Molecular biology program, Université de Montréal, P.O. Box 6128, Succursale Centre-ville, Montreal, H3C 3J7, Canada; 3 Infectious Diseases Research Centre-CRI, CHU de Québec Research Center (CHUQ), Université Laval, Québec, G1V 4G2, Canada; 4 Department of Microbiology-Infectious Disease and Immunology, Faculty of Medicine, Université Laval, Québec, G1V 0A6, Canada; 5 Division of Biomedical and Life Sciences, Faculty of Health and Medicine, Lancaster University, Lancaster, LA1 4YQ, UK; 6 Department of Medicine, Université de Montréal, P.O. Box 6128, Succursale Centre-ville, Montreal, H3C 3J7, Canada

## Abstract

The mechanism of action of valproate (VPA), a widely prescribed short chain fatty acid with anticonvulsant and anticancer properties, remains poorly understood. Here, the yeast *Saccharomyces cerevisiae* was used as model to investigate the biological consequences of VPA exposure. We found that low pH strongly potentiates VPA-induced growth inhibition. Transcriptional profiling revealed that under these conditions, VPA modulates the expression of genes involved in diverse cellular processes including protein folding, cell wall organisation, sexual reproduction, and cell cycle progression. We further investigated the impact of VPA on selected processes and found that this drug: i) activates markers of the unfolded protein stress response such as Hac1 mRNA splicing; ii) modulates the cell wall integrity pathway by inhibiting the activation of the Slt2 MAP kinase, and synergizes with cell wall stressors such as micafungin and calcofluor white in preventing yeast growth; iii) prevents activation of the Kss1 and Fus3 MAP kinases of the mating pheromone pathway, which in turn abolishes cellular responses to alpha factor; and iv) blocks cell cycle progression and DNA replication. Overall, our data identify heretofore unknown biological responses to VPA in budding yeast, and highlight the broad spectrum of cellular pathways influenced by this chemical in eukaryotes.

Sodium valproate (or valproic acid; VPA) is a short chain fatty acid with anticonvulsant properties and low toxicity that is widely prescribed to treat seizures, schizoaffective disorders, and migraines, although the molecular basis of its therapeutic efficacy is unclear^1^. VPA influences a broad spectrum of cellular processes in part by inhibiting class I and II histone deacetylases (HDACs)^2^,^3^. HDACs reverse lysine acetylation on histones, thereby influencing gene expression by promoting a repressive chromatin state^4^. HDACs also deacetylate lysines in non-histone proteins^5^ which adds considerable complexity to the effects of HDAC inhibition. Since cancer cells are well-known to present epigenetic defects, VPA has garnered interest as a potential anticancer drug^6^. VPA inhibits growth and cell cycle progression, and induces apoptosis and senescence, in cancer cells *in vitro*^7^ and in xenograft models^8^. Clinical trials also revealed that VPA synergizes with genotoxic chemotherapy drugs, e.g. doxorubicin^9^ and cisplatin^10^, in mitigating cancer cell growth. Nevertheless, the biological responses to VPA remain to be fully elucidated.

Yeast is a model organism of choice to investigate the mechanism of action of pharmacological agents. As for human cells, protracted VPA exposure causes apoptosis in the budding yeast *Saccharomyces cerevisiae*^11^,^12^. VPA-mediated HDAC inhibition also leads to hyperacetylation of certain DNA repair proteins in this organism, resulting in their autophagic degradation and consequent DNA repair defects^13^. In the fission yeast *Schizosaccharomyces pombe*, genomic screens identified genes involved in membrane trafficking (retromer complex) and signal transduction (Ras, MAP kinases) whose mutation cause hypersensitivity to VPA^14^,^15^,^16^. VPA also perturbs the cell wall and generates synergistic growth defects when combined with cell wall stress-causing treatments, e.g. zymolyase and micafungin, in *S. pombe*^16^. Consistent with the fact that cell wall and membrane/endoplasmic reticulum perturbations elevate cytoplasmic calcium^17^,^18^, VPA also induces extracellular Ca^2+^ influx in *S. pombe*^15^. Finally, recent reports indicate that VPA perturbs inositol metabolism in *S. cerevisiae*, leading to defects in vacuolar function^19^. Overall the available data suggest that VPA induces complex cellular responses in fungi as well as in human cells.

Here, we used *S. cerevisiae* to investigate the consequences of VPA exposure on gene transcription, cell signalling, and proliferation. We found that at low environmental pH, the toxicity and biological effects of VPA are strongly potentiated, and that several important cellular pathways are influenced by VPA in these conditions. Our work identifies and characterizes previously unreported biological responses to VPA in *S. cerevisiae*, and highlights the broad spectrum of cellular pathways influenced by this chemical in eukaryotic cells.

## Results

### VPA inhibits cell proliferation in a pH-dependent manner

**Figure 1:**
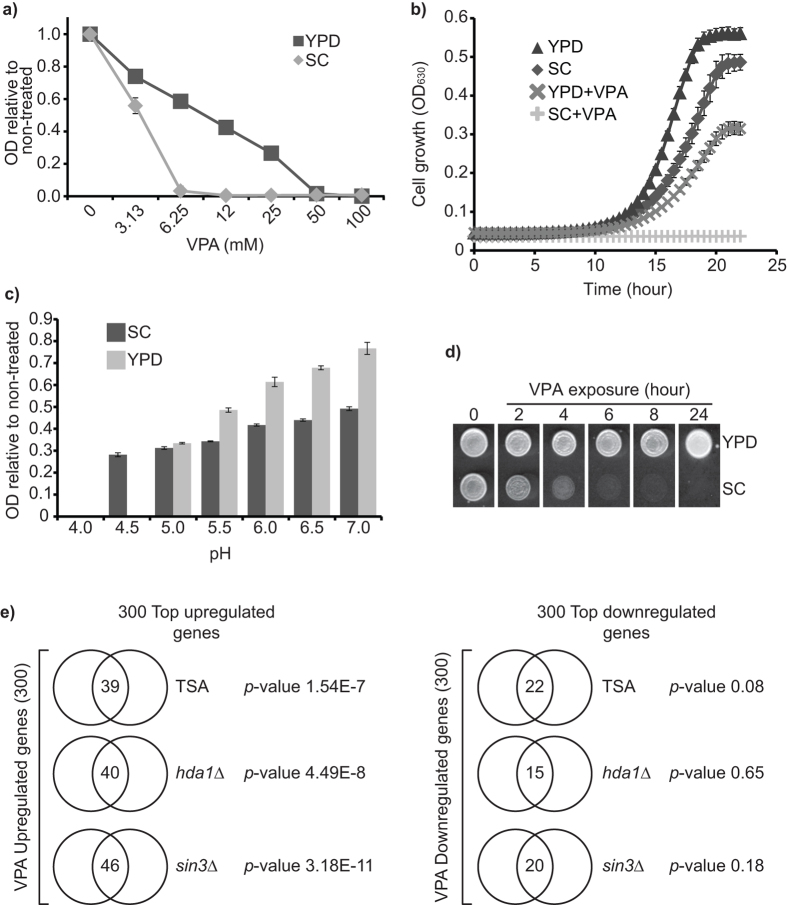
VPA inhibits cell proliferation in a pH-dependent manner and generates a complex transcriptional response which partly reflects its HDAC-inhibiting properties. (**a**) VPA inhibits cell proliferation at lower concentrations in SC compared to YPD medium. Cell growth in VPA was monitored by OD_630_ measurements after 48 h. (**b**) 10 mM VPA inhibits cell proliferation in SC medium. OD_630_ was monitored every 30 minutes for 24 h. (**c**) VPA-induced growth inhibition depends on environmental pH. The pH of SC and YPD was adjusted with hydrochloric acid or sodium hydroxide. Cell growth was assessed as in A. (**d**) VPA is fungicidal in SC. Yeast cultures were incubated in SC or YPD containing 10 mM VPA at 30 °C. Cells were spotted on YPD-agar after the indicated time, and plates were incubated at 30 °C. (**e**) VPA-induced transcriptional changes present similarity to those elicited by trichostatin A (TSA) or deletion of HDAC-encoding genes (*sin3*Δ or *hda1*Δ). The top 300 up- and downregulated genes in response to VPA were compared to published datasets using Venn diagrams; p-values were calculated using hypergeometric tests (see Methods).

VPA displayed more potent growth-inhibiting properties in synthetic (SC) as compared to rich (YPD) medium (Fig. 1a,b). The pH of SC was 4.1, whereas that of YPD is 6.8. Since VPA is a weak acid (pKa 4.8), charge neutralization at low pH could facilitate diffusion of the drug across membranes and increase its biological activity. Consistently, the antiproliferative properties of VPA sharply increase as pH decreases from 5 to 4 in either YPD or SC (Fig. 1c). Yeast cells incubated in SC containing VPA for 2 hours exhibited loss of viability, whereas no such effect was observed in YPD (Fig. 1d). These data indicate that low environmental pH influences VPA-induced growth inhibition in budding yeast.

### VPA induces a complex transcriptional response that only partly reflects its HDAC inhibiting properties

We hypothesized that the observed pronounced biological effects of VPA at low pH resulted from increased HDAC inhibition^2^,^3^. Yeast lacking archetypal class I and II HDACs, e.g. Hda1 and Rpd3, present hyperacetylated lysines in histones (H3K9, H4K5, H4K12) at specific genomic regions^20^,^21^. However, immunoblotting revealed that global acetylation at these residues is not noticeably modulated by exposure to 10 or 50 mM VPA, or to 50 mM of the structurally-similar HDAC inhibitor Sodium Butyrate, for up to four hours in SC at pH 4 or 7 (Supplementary Fig. S1). Immunoblots using anti-H4K16ac or anti-acetyllysine antibodies also did not reveal pH-dependent effects of VPA on protein acetylation (Supplementary Fig. S1). HDAC inhibition does not sensitize cells to low pH, since none of the HDAC deletion strains tested presented growth defects at pH 4 (Supplementary Fig. S1). These data suggest that pH-dependent effects of VPA on cell proliferation are unlikely attributable to increased VPA-induced HDAC inhibition. However, it remains possible that VPA influences histone acetylation at specific genomic loci, or on a limited number of non-histone proteins.

Since HDACs influence gene expression^4^, we performed RNA-seq to document transcriptional changes elicited by 60 minutes exposure to VPA in pH 4.1 SC medium (Supplementary Table S1). Using a cut-off of two-fold difference in expression, 297 and 300 genes were up- and downregulated by VPA, respectively (Supplementary Table S1). We compared our data with those generated in response to Trichostatin A (TSA), a class I/II HDAC inhibitor, or in *sin3∆* (a component of Rpd3-containing HDAC complexes) or *hda1*∆ mutants (see Methods). A limited but significant number of VPA-upregulated genes are also upregulated by TSA and *sin3∆* or *hda1∆* mutations, but no significant overlap was detected for downregulated genes (Fig. 1e, Supplementary Table S2). These limited similarities suggest that VPA can probably influences gene expression in HDAC-independent manners.

To identify cellular processes influenced by VPA, Gene Ontology (GO)-term analysis was performed. Genes involved in metabolism (e.g. amino acid biosynthesis, alcohol and glucose catabolic processes, etc.), protein folding, response to abiotic stimulus, post-transcriptional regulation of gene expression, and copper import were upregulated, while genes involved in cell morphogenesis, carbohydrate transport, cell cycle (e.g. cell division, M phase, DNA replication), reproductive cellular processes, amino acid transport, and cell wall organisation were downregulated (Table 1). This transcriptional signature is consistent with known effects of VPA in fungi. Endoplasmic reticulum/membrane trafficking functions are influenced by VPA in budding and fission yeast^15^,^16^,^19^, which is concordant with transcriptional modulation of protein folding processes. VPA compromises cell wall structure^16^ and may influence MAP kinase signalling^15^ in *S. pombe*, which could explain modulation of cell wall organisation and reproductive processes genes since these pathways are controlled by MAP kinases^22^,^23^. Finally, VPA-induced growth inhibition at reduced pH (Fig. 1) may explain downregulation of cell cycle genes. With this in mind, we selected a subset of VPA-influenced processes for further characterization, i.e. protein folding, cell wall organisation, reproduction/mating, and cell cycle progression.

### VPA activates the unfolded protein response

**Figure 2:**
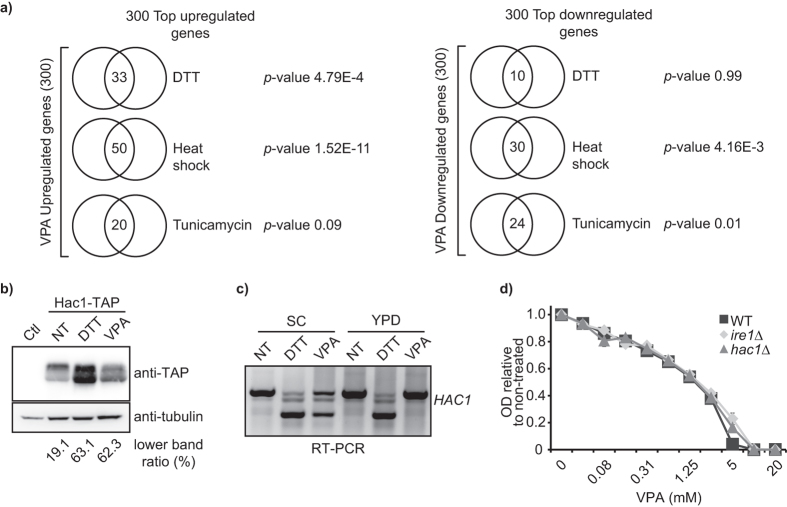
VPA elicits markers of the canonical UPR pathway in *S. cerevisiae*. (**a**) VPA-induced transcriptional changes present similarity to those elicited by treatments causing unfolded protein stress. The top 300 up- and downregulated genes in response to VPA were compared to published datasets using Venn diagrams; p-values were calculated using hypergeometric tests (see Methods). (**b,c**) VPA causes splicing of *HAC1* mRNA and expression of the short isoform of Hac1. Cells were incubated in SC medium +/−10 mM VPA or 6 mM DTT for one hour. Samples were processed for immunoblotting (**b**), or total RNA was extraction followed by for RT-PCR (**c**). The ratio of the intensity of lower band on the total intensity of the two *HAC1* bands (upper and lower) is presented in %. NT: non-treated (**d**) Cells lacking the key UPR factors Hac1 or Ire1 are not hypersensitive to VPA. Cell growth in VPA was monitored by OD_630_ measurements after 48 h.

We noted similarities between the VPA transcriptional signature and that of the unfolded protein stress response (UPR)^24^,^25^ (Supplementary Table S1, Table 1). The UPR reduces the load of proteins entering the endoplasmic reticulum (ER), and promotes degradation or secretion of unfolded proteins^26^. In response to UPR stressors such as dithiothreitol (DTT), tunicamycin, or heat shock, fungi modulate the expression of genes related to vesicle trafficking, protein folding, amino acid metabolism, proteolysis, glycosylation, lipid metabolism, and cell wall biogenesis^27^,^28^. Except for glycosylation genes, VPA induced a similar signature (Supplementary Table S3). The largest fraction of VPA-responsive transcripts related to UPR belongs to heat shock proteins with chaperone activity, amino acid and lipid metabolism (Supplementary Table S3). We also found significant overlap between genes that are upregulated in response to VPA vs DTT or heat shock, but not tunicamycin (Fig. 2a, Supplementary Table S2, see Methods), and between genes that are downregulated in response to VPA vs heat shock or tunicamycin. We conclude that VPA-induced transcriptional changes include genes that are typically modulated by UPR-inducing conditions.

Upon canonical UPR activation, the transmembrane protein kinase/nuclease Ire1 promotes splicing of *HAC1* mRNA, leading to the expression of a shorter, more stable form of the Hac1 transcription factor and upregulation of UPR genes^26^. *ire1∆* and *hac1∆* mutants are sensitive to several UPR-inducing conditions^29^, although Ire1- and Hac1-independent pathways also influence gene expression upon unfolded protein stress^30^. Exposure to VPA in SC stimulated the expression of the shorter isoform of Hac1 and splicing of the *HAC1* mRNA, albeit to a reduced extent compared to DTT (Fig. 2b,c). However, deletion of *HAC1* or *IRE1* did not cause hypersensitivity to VPA (Fig. 2d). Our data therefore indicate that even though the Hac1/Ire1-mediated canonical UPR is not necessary for survival in response to VPA, this drug elicits typical UPR markers in budding yeast.

### VPA causes influx of extracellular calcium

**Figure 3:**
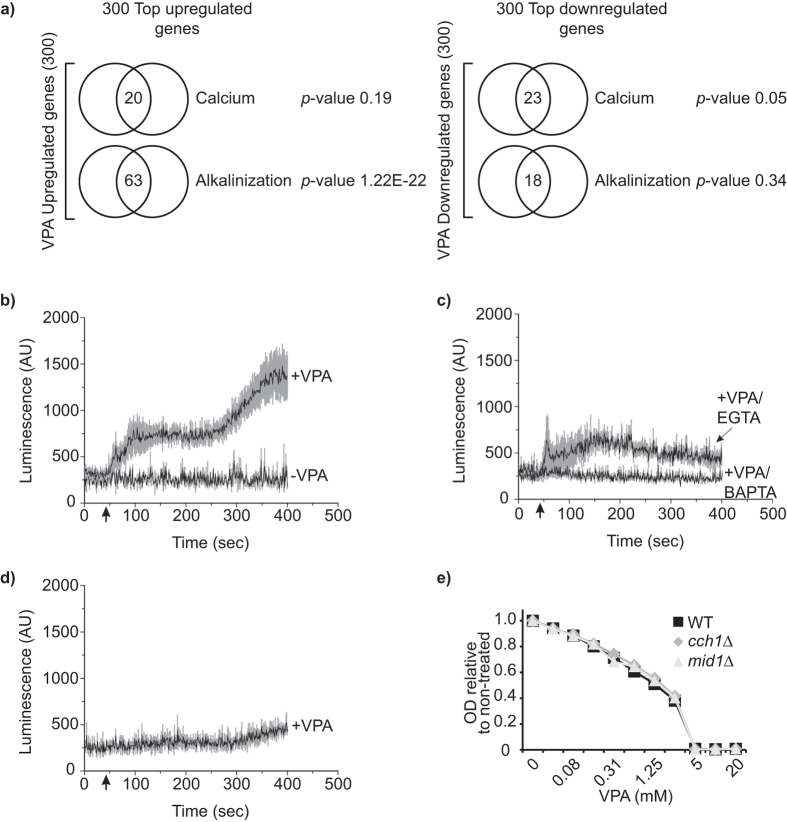
VPA causes influx of extracellular calcium via the Cch1/Mid1 channels in *S. cerevisiae.* (**a**) Similarities between VPA-induced transcriptional changes and those caused by medium alkalinization. The top 300 up- and downregulated genes in response to VPA were compared to published datasets using Venn diagrams; p-values were calculated using hypergeometric tests (see Methods). (**b–d**) Valproic acid induces Cch1-mediated cytoplasmic [Ca^2+^] elevation. Ca^2+^-dependent aequorin luminescence was measured in WT (**b,c**) or *cch1*∆ (d) cells in response to 8 mM valproic acid (VPA). VPA was added in SC medium (+VPA), SC containing 2 mM EGTA (+VPA/EGTA), or in BAPTA buffer (+VPA/BAPTA). Results represent mean (± standard error of the mean; SEM) from at least 4 independent experiments. SEM values are illustrated with grey shading. Luminescence was recorded every second and is expressed in arbitrary units (AU). (**e**) Cells lacking the Cch1/Mid1 channels are not hypersensitive to VPA. Cell growth in VPA was monitored by OD_630_ measurements after 48 h.

Cell survival under conditions that compromise protein folding rely in part on Ca^2+^ influx and activation of Calcineurin, a calcium-responsive phosphatase^31^. Since VPA causes calcium influx in *S. pombe*^15^, we compared VPA-induced transcriptional changes with those resulting from medium alkalinisation and extracellular CaCl_2_, two conditions known to elevate cytosolic Ca^2+^. We found significant overlap between genes that are up-, but not down-regulated in response to medium alkalinisation vs VPA, but no significant overlap for either up- or down-regulated genes in the case of elevated extracellular calcium concentrations (Fig. 3a, Supplementary Table S2, see Methods). Since medium alkalinisation induces a broad spectrum of cellular responses in addition to calcium signalling^32^, we sought to directly investigate whether VPA influences calcium homeostasis in budding yeast. We transformed cells with the pEVP11/AEQ plasmid which expresses the Ca^2+^-sensitive bioluminescent protein aequorin. VPA increased cytoplasmic Ca^2+^ in *S. cerevisiae* grown in SC (Fig. 3b), although the effect was modest (less than 5-fold increase over resting levels) compared to *S. pombe* (>200-fold over resting levels^15^). The observed effect depends on Ca^2+^ influx across the plasma membrane since addition of EGTA or BAPTA (which both chelate Ca^2+^) to the medium inhibited cytoplasmic Ca^2+^ elevation (Fig. 3c). In *S. pombe,* VPA-induced Ca^2+^ influx requires the Cch1-Yam8 voltage-gated high-affinity calcium channel^15^, encoded in *S. cerevisiae* by *CCH1* and *MID1*^33^. VPA-induced cytoplasmic Ca^2+^ accumulation was abrogated in *cch1*Δ mutants (Fig. 3d). However, *cch1*∆ and *mid1∆* mutants were not hypersensitive to VPA in SC (Fig. 3e), as was the case for calcineurin (e.g. *cna1∆, cnb1∆, cmp2∆)* or calmodulin mutants (e.g. *cmk1*Δ, *cmk2*Δ; Supplementary Fig. S2). Overall, our results indicate i) that calcium influx is not required for survival of budding yeast in response to VPA, and ii) that the transcriptional signature induced by this chemical presents little similarities with that resulting from known cytoplasmic calcium-elevating treatments, e.g. high extracellular calcium concentrations. The biological significance of VPA-induced elevation of cytoplasmic Ca^2+^ in *S. cerevisiae*, if any, therefore remains unresolved.

### VPA inhibits activation of the Slt2 cell wall integrity (CWI) MAP kinase

**Figure 4:**
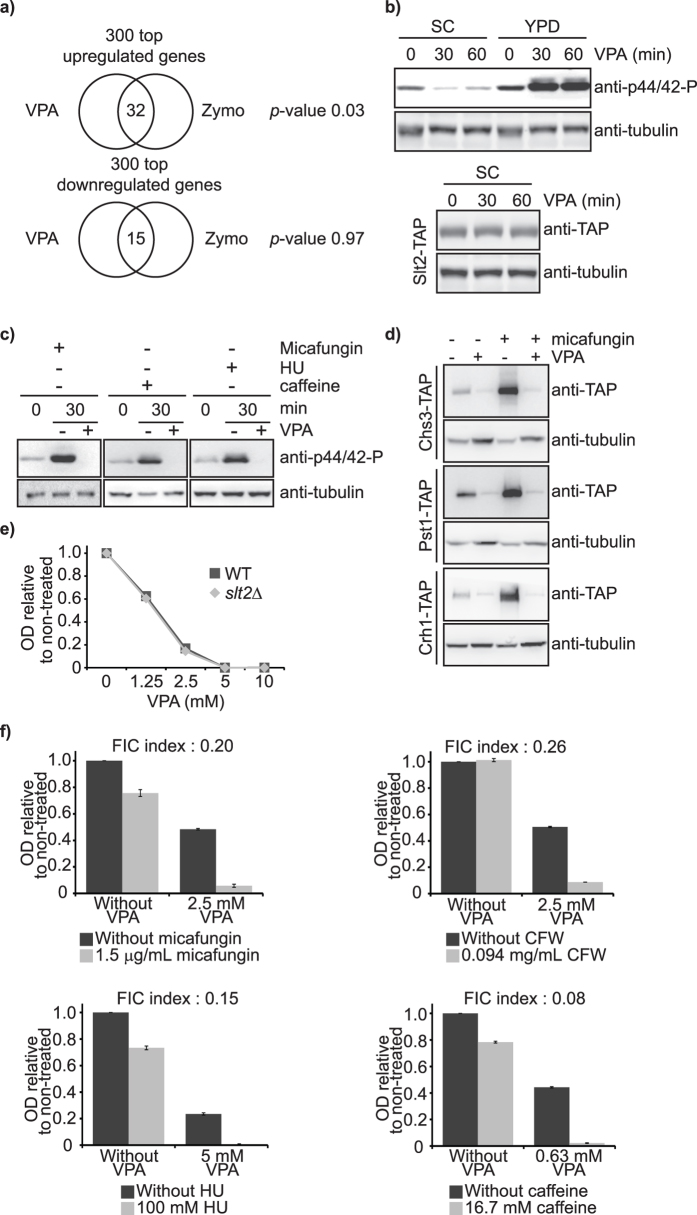
VPA modulates the cell wall integrity (CWI) pathway by inhibiting the Slt2 MAP kinase. (**a**) Similarities between VPA-induced transcriptional changes and those caused by zymolyase. The top 300 up- and downregulated genes in response to VPA were compared to published datasets using Venn diagrams; p-values were calculated using hypergeometric tests (see Methods). (**b**) VPA inhibits basal Slt2 phosphorylation in SC but not in YPD medium. Cells were incubated with 10 mM VPA in SC or YPD, and samples were processed for immunoblotting with an anti-phosphorylated human p44/42 antibody (Top panel). Cells expressing Slt2-TAP were processed as above, but with an anti-TAP antibody (Bottom Panel). (**c**) VPA inhibits stress-induced phosphorylation of Slt2. Cells were incubated for 30 minutes in SC +/−10 mM VPA, and 2 μg/mL micafungin, 200 mM hydroxyurea (HU), or 15 mM caffeine. (**d**) VPA inhibits micafungin-induced expression of Rlm1 target genes. Cells were incubated for 30 minutes in SC +/−10 mM VPA and 2 μg/mL micafungin. Samples were prepared for immunoblotting with an anti-TAP antibody. (**e**) *slt2∆* mutants are not hypersensitive to VPA. Cell growth in VPA was monitored by OD_630_ measurements after 48 h. (**f**) VPA synergizes with drugs that activate the CWI pathway in preventing cell growth. Cell growth was monitored as in (**e**). FIC indexes were calculated as described in Methods. CFW: calcofluor white.

VPA compromises cell wall integrity in fission yeast^16^. Expression of cell wall organisation genes is influenced by VPA in budding yeast (Table 1), while significant overlap was observed between genes upregulated in response to VPA and those upregulated in response to zymolyase-mediated cell wall digestion (Fig. 4a, Supplementary Table S2, see Methods). We therefore investigated the influence of VPA on CWI signalling. The Slt2 MAP kinase is activated by phosphorylation in response to cell wall perturbation caused by micafungin, or upon exposure to caffeine or hydroxyurea; however these latter two agents, unlike micafungin, influence several cellular pathways and thus are not specific activators of CWI signalling^22^,^34^,^35^. Unexpectedly, VPA abolished basal levels of Slt2 phosphorylation in SC (as detected by an antibody directed against human phospho-p44/42 ERK kinase), whereas the opposite was observed in YPD (Fig. 4b, top panel). This was not due to VPA-induced reduction in Slt2 protein levels (Fig. 4b, bottom panel). Expression of genes acting upstream of Slt2 in the CWI cascade was downregulated by VPA (e.g. *WSC2, ROM1, BCK1*; Supplementary Table S4), suggesting that transcriptional repression of upstream activators might contribute to VPA-induced inhibition of Slt2 phosphorylation. Induction of Slt2 phosphorylation by micafungin, hydroxyurea, or caffeine was also abolished by VPA in SC (Fig. 4c). CWI signalling culminates with activation of the Rlm1 transcription factor, and expression of its target genes^22^,^36^. VPA abrogated micafungin-induced expression of three proteins encoded by such genes, i.e. Crh1, Pst1, Chs3 (Fig. 4d), even though *RLM1* expression was not modulated by VPA (Supplementary Table S4). We also verified whether VPA promotes Slt2 dephosphorylation. Contrary to this notion, neither genes encoding Slt2 phosphatases^35^,^37^,^38^ (*SDP1, MSG5, PTP2, PTP3*), nor ones that dephosphorylate its activating kinases Mkk1/2^39^,^40^ (*PTC1, PTC6*), were upregulated by VPA (Supplementary Table S1). Consistently, VPA-induced abrogation of Slt2 phosphorylation was observed in cells devoid of the aforementioned phosphatases (Supplementary Fig. S3).

Cells lacking Slt2 are sensitive to cell wall stressors such as micafungin and calcofluor white, and also to other Slt2-activating compounds, e.g. caffeine and hydroxyurea^22^,^34^,^35^. Slt2 deficiency did not modulate sensitivity to VPA in SC (Fig. 4e). Interestingly, VPA synergized (fractional inhibitory concentration^41^ (FIC) index < 0.5) with micafungin, calcofluor white, caffeine, and hydroxyurea in inhibiting budding yeast growth (Fig. 4f). Our results are therefore consistent with a model whereby VPA causes both cell wall stress^16^ and downregulation of Slt2 activity, which may contribute to VPA-mediated growth-inhibition.

### VPA inhibits the mating pheromone pathway

**Figure 5:**
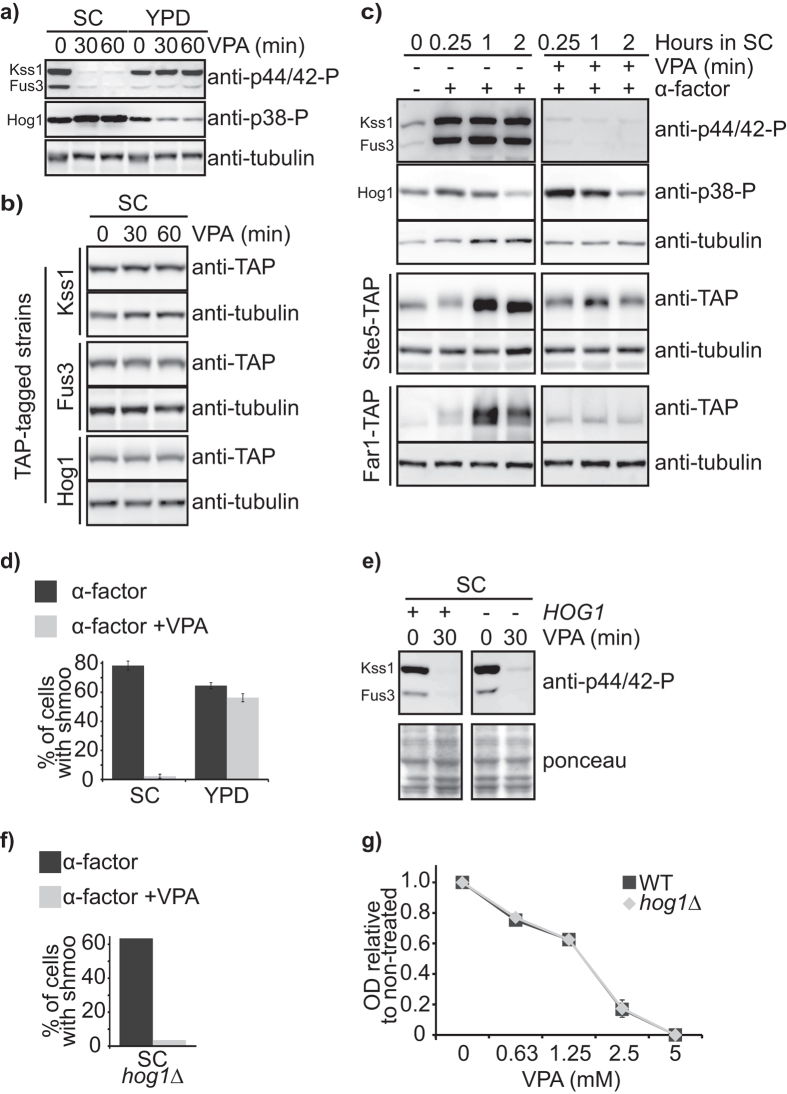
VPA inhibits the mating pheromone signaling pathway. (**a**) VPA inhibits basal phosphorylation of MAP Kinases of the pheromone pathway in SC but not in YPD medium. Cells were treated with 10 mM VPA in the indicated medium. Samples were processed for immunoblotting with an anti-tubulin antibody as loading control, anti-phospho human p44/42 antibody that recognizes phosphorylated Kss1 and Fus3, or anti-phospho human p38 antibody recognizing phosphorylated Hog1. (**b**) VPA does not influence Kss1, Fus3, or Hog1 protein levels. Yeast strains expressing TAP-tagged versions of the indicated proteins were incubated in SC containing VPA. Samples were processed for immunoblotting with an anti-TAP (CBP) antibody. (**c**) VPA inhibits the alpha factor mating pheromone response. Cells were incubated in SC with or without alpha factor and VPA. Samples were processed for immunoblotting as in (**a,b**). (**d**) VPA inhibits alpha factor-induced shmoo formation in SC but not in YPD. Cells were incubated in the indicated media for 2 hours. Samples were examined by microscopy to monitor shmoo formation. (**e,f**) VPA inhibits Kss1 and Fus3 phosphorylation in a *HOG1*-independent manner. (**e**) Cells were incubated in SC containing VPA. Samples were processed for immunoblotting as in (**a**). (**f**) VPA inhibits shmoo formation in a *HOG1*-independent manner. Cells were treated as in (**d**). (**g**) *hog1∆* cells are not hypersensitive to VPA. Cell growth in VPA was monitored by OD_630_ measurements after 48 h.

VPA-induced transcriptional changes include downregulation of genes controlling reproductive cellular processes (Table 1). The human anti-phospho-p44/42 ERK used in Fig. 4 also recognizes phosphorylated forms of the Fus3 and Kss1 MAP kinases which regulate the alpha factor mating pheromone pathway^42^. In SC, but not YPD, VPA abrogated basal and alpha factor-induced Kss1 and Fus3 phosphorylation, without affecting levels of these proteins (Fig. 5a–c). Consistently, alpha factor-induced phosphorylation (detected by electrophoretic mobility shift) and upregulation of Far1 (a downstream target of Fus3) and of the Ste5 scaffolding protein (which permits sequential phosphorylation of kinases of the mating pheromone cascade), was impaired by VPA in SC (Fig. 5c), as was alpha factor-induced shmoo formation (Fig. 5d).

We hypothesized that VPA-induced modulation of gene expression renders cells pseudo-diploid, thereby preventing responses to alpha factor. Expression of a modest number of diploid-specific genes was influenced by VPA (Supplementary Fig. S4, Supplementary Table S5, see Methods), only a few of which directly regulate the mating pheromone pathway (e.g. PRM1, FUS2, FAR1, BUD3)^43^. Moreover these factors act downstream of Kss1 and Fus3, and as such their downregulation appears unlikely to influence phosphorylation of these MAP kinases. *STE20* is the only gene encoding a factor acting upstream of Kss1 and Fus3 that is modestly repressed by VPA (1.8-fold; Supplementary Table S5). VPA-induced transcriptional modulation is therefore unlikely to contribute significantly to the inhibitory effect of VPA on the mating pheromone pathway.

Activation of the Hog1 osmotic stress kinase inhibits alpha factor signalling^44^. VPA exposure in SC, but not in YPD, modestly increased Hog1 phosphorylation (Fig. 5a,c). However, deletion of *HOG1* did not prevent VPA-induced inhibition of Kss1 and Fus3 phosphorylation (Fig. 5e), and VPA-treated *hog1∆* cells did not form shmoos in response to alpha factor (Fig. 5f), suggesting that Hog1 does not mediate VPA-induced inhibition of the mating pheromone pathway. Moreover the osmoresponsive pathway is not required for growth in VPA since *hog1∆* cells are not hypersensitive to this drug (Fig. 5g).

### VPA inhibits cell cycle progression

**Figure 6:**
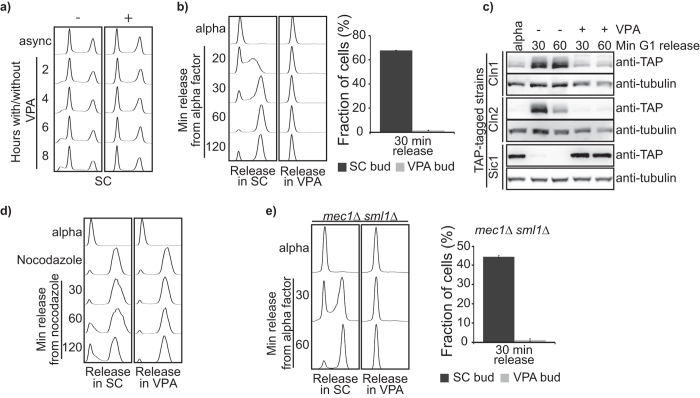
VPA inhibits the G1 to S and G2/M to G1 transitions. (**a**) VPA-treated cells do not accumulate in a particular phase of the cell cycle. Cells were incubated in SC medium containing 10 mM VPA. Samples were processed for DNA content analysis by flow cytometry. Async: asynchronously growing cells. (**b**) VPA inhibits the G1 to S transition. Cells were synchronized in G1 with alpha factor and released in SC +/−VPA. Samples were processed for DNA content analysis by flow cytometry (left panel) or examined by microscopy to assess budding (right panel). (**c**) VPA inhibits Cln1-2 expression and Sic1 downregulation during the G1 to S transition. Cells were synchronized in G1 and released in SC +/−VPA. Samples were processed for immunoblotting with an anti-TAP (CBP) or anti-tubulin antibody. (**d**) VPA inhibits progression from G2/M to G1. Cells were synchronized in G1 and released toward S in nocodazole for 90 minutes. Cells were then incubated in SC +/−VPA. Samples were processed for DNA content analysis by flow cytometry. (**e**) Mec1 is not required for VPA-induced inhibition of cell cycle progression and shmoo formation. Cells were treated as in (**b**). Samples were processed for DNA content analysis by flow cytometry (left panel) or examined by microscopy to assess budding (right panel).

Since VPA causes downregulation of cell cycle genes (Table 1) and growth inhibition, we characterized the impact of this drug on cell cycle progression. In SC medium, VPA did not cause obvious accumulation of cells in any particular phase of the cell cycle (Fig. 6a). Since cells do not proliferate under these conditions (Fig. 1), the results suggest that VPA inhibits transition between cell cycle phases. We investigated whether VPA influences the G1 to S transition by synchronizing cells in G1 with alpha factor and releasing them in VPA-containing SC. Strikingly, VPA-treated cells did not form buds and their DNA content did not increase two hours after release from G1 (Fig. 6b). The G1-S transition depends on cyclin-dependent kinase activity and on Cln1/2 expression^45^. We found that Cln1 and Cln2 are not expressed when cells are released from G1 in VPA-containing SC (Fig. 6c). Consistently, mRNA levels for the corresponding genes were downregulated upon VPA exposure (Supplementary Table S1), and expression of the Clb5/Clb6-CDK inhibitor Sic1, whose stability is downregulated in a Cln1/2-CDK manner^46^, was maintained in cells released from G1 in VPA-containing SC (Fig. 6c). We conclude that downregulation of Cln1/2 expression probably contributes to VPA-induced G1 to S transition defects. We also found that nocodazole-arrested G2/M cells released in VPA-containing SC failed to complete mitosis and to initiate the next G1 (Fig. 6d), although the mechanism remains to be elucidated.

During genotoxic stress in yeast, activation of the DNA damage checkpoint kinases Mec1 and Rad53 inhibits Cln1/2 expression and the G1-S transition^47^,^48^. We hypothesized that VPA might cause DNA damage, thereby inducing a Mec1-dependent checkpoint response. Contrary to this idea, VPA inhibited the G1-S transition and prevented bud formation in cells lacking *MEC1* (Fig. 6e). We conclude that DNA damage checkpoint activation is unlikely to contribute to VPA-mediated inhibition of the G1-S transition.

### VPA inhibits DNA replication

**Figure 7:**
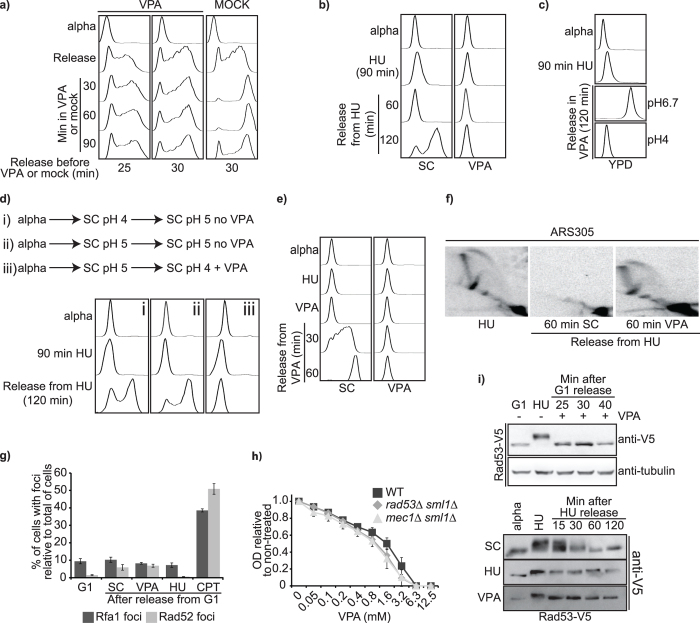
VPA inhibits DNA replication without causing DNA damage. (**a**) VPA inhibits DNA replication. Cells were synchronized in G1 using alpha factor and released toward S for 25 or 30 minutes before addition of VPA. Samples were processed for DNA content analysis by flow cytometry. (**b**) VPA inhibits resumption of DNA replication after hydroxyurea (HU) treatment. Cells were synchronized in G1, released in SC containing 200 mM HU for 90 minutes, and then incubated in SC +/−VPA. (**c**) VPA inhibits S phase progression in pH4 YPD. Cells were processed as in (**b**). (**d**) VPA-induced pH changes do not influence DNA replication. Cells were synchronized in G1 at pH 4.1 (left panel) or pH 5 (middle and right panel). Cells were released toward S (at the same pH) in HU for 90 minutes. Cells were then incubated in SC pH 5 (left and middle panel), or in SC with VPA (right panel; resulting pH is 5). (**e**) VPA reversibly inhibits DNA replication. Cells were treated as in B, but incubated in SC after VPA. (**f**) VPA does not influence the structure of HU-stalled DNA replication forks. 2D gel electrophoresis was performed as described in Methods. (**g**) VPA does not cause Rad52 or Rfa1 foci. Cells expressing Rad52-YFP or Rfa1-8ala-YFP were synchronized in G1 and released toward S in SC for 30 minutes, followed by addition of VPA for 60 minutes. As controls, cells were released in SC with 5 μg/mL camptothecin (CPT) or 200 mM HU. Samples were examined by fluorescence microscopy. (**h**) Lack of Mec1 or Rad53 does not sensitize to VPA. OD_630_ was monitored after 48 h. (**i**) VPA does not influence Rad53 phosphorylation. Left panel: Cells were synchronized in G1 and released toward S in SC. VPA was added after 25, 30 or 40 minutes, and cells were further incubated for 60 minutes. As control, cells were released from G1 in HU for 60 minutes. Right panel: Cells expressing Rad53-V5-6His were synchronized in G1 and released toward S in HU for 60 minutes. Cells were washed and incubated in SC +/−VPA or HU.

The fraction of S phase cells in asynchronous yeast populations is relatively low, precluding us from ascertaining whether VPA influences DNA replication (Fig. 6a). To circumvent this, cells were arrested in G1 with alpha factor and released toward S in SC for 25 or 30 minutes before addition of VPA. Strikingly, under these conditions, VPA completely blocked DNA replication (Fig. 7a). VPA rapidly blocks active DNA replication forks since replication does not progress between the time of VPA addition and subsequent time points. We next arrested cells in G1 and released them toward S in the presence of hydroxyurea (HU) or methyl methanesulfonate (MMS), two DNA replication-blocking drugs, to synchronize them in early- and -mid S phase, respectively, before VPA addition (Fig. 7b, Supplementary Fig. S5). More than 95% of cells present buds under these conditions, indicating S phase entry (data not shown). Removal of HU or MMS followed by incubation in SC allowed cells to complete DNA replication within 60 to 120 minutes. In contrast, DNA content of cells incubated in SC containing VPA did not change over this period (Fig. 7b, Supplementary Fig. S5), indicating that VPA inhibits resumption of DNA replication after HU- or MMS-induced arrest in yeast.

We tested whether VPA-mediated inhibition of DNA replication depends on pH. Addition of VPA did not prevent DNA replication in YPD at pH 6.7 (Fig. 7c), although elevated drug concentrations delayed replication to some extent (Supplementary Fig. S5). In contrast, VPA completely inhibited DNA replication in YPD at pH 4 (Fig. 7c). pH measurements revealed that addition of 10 mM VPA to SC medium increased its pH to 5, raising the possibility that VPA-induced inhibition of DNA replication could result from a sudden increase in pH. However, releasing cells from alpha factor arrest into pH4 SC medium and then raising the pH to 5 to simulate addition of VPA (Fig. 7d panel i) did not influence DNA replication. Moreover, maintaining a constant pH of 5 throughout the experiment did not abrogate the effect of VPA on replication (Fig. 7d, compare panel ii and iii). Activation of the Hog1 kinase delays S phase progression via phosphorylation of the Mrc1 replisome protein^49^. We hypothesized that VPA-induced Hog1 activation (Fig. 5a) might inhibit DNA replication. Contrary to this notion, VPA blocked completion of DNA replication in *hog1∆* cells (Supplementary Fig. S6). Finally, VPA-induced autophagy^20^ and extracellular calcium influx also did not impact DNA replication (Supplementary Figs S2, S7).

### VPA does not compromise the integrity of DNA replication forks

We hypothesized that VPA could irreversibly compromise active DNA replication forks. In disagreement with this, cells rapidly resume DNA replication after removal of VPA from the medium (Fig. 7e), and neutral two-dimension gel electrophoresis^50^ revealed that DNA structures formed at the ARS 305 early replication origin after 90 minutes in HU were unchanged upon incubation in VPA (Fig. 7f). Camptothecin (CPT) impedes DNA replication and causes DNA lesions that are processed by homologous recombination (HR). Treatment with CPT, but not with HU, causes focus formation of the HR factors Rad52 and Rfa1 during S phase^51^. To evaluate whether VPA is genotoxic, cells were synchronized in G1, and released in SC containing either HU or CPT as controls, or in SC for 30 minutes followed by addition of VPA. As expected, CPT treatment yielded high frequencies of Rad52 or Rfa1 foci. In contrast, exposure to HU or VPA did not cause focus formation for either protein (Fig. 7g). Even though HU does not generate HR foci in S phase cells, this drug causes loss of viability in *mec1∆ sml1∆* and *rad53∆ sml1∆* mutants which are exquisitely sensitive to perturbations of DNA replication^52^. Consistent with the notion that VPA does not compromise DNA replication forks, cells lacking Mec1 or Rad53 were not hypersensitive to VPA in SC (Fig. 7h). Upon HU-induced replicative stress, Mec1 phosphorylates its downstream target Rad53^52^. Cells exposed to VPA during S phase did not exhibit Rad53 phosphorylation (Fig. 7i top panel), as assessed by phosphorylation-induced electrophoretic mobility shift. VPA also did not cause Rad53 phosphorylation to persist after removal of HU from the medium (Fig. 7i bottom panel). Overall, our results are concordant with a model whereby VPA is not genotoxic in yeast, and therefore blocks active DNA replication fork via a DNA damage-independent mechanism.

## Discussion

Here, we used budding yeast as model to investigate the biological consequences of exposure to VPA, a widely prescribed antiepileptic^1^ drug and potential anticancer^6^ agent. We found that low pH strongly potentiated the growth-inhibiting properties of VPA, in accord with published studies documenting pH-dependent VPA-induced growth inhibition and apoptosis in *S. pombe*^53^. VPA-induced apoptosis has also been reported in *S. cerevisiae,* although these data were generated using YPD medium (which is normally at higher pH than SC) over long time periods, i.e., 24 h or more^11^,^12^. Yca1 (a caspase-like protease) and the class III HDAC Sir2 are required for apoptosis in response to VPA in budding yeast^11^,^12^. While we did not formally test whether VPA caused apoptosis under our conditions, deletion of *YCA1* and *SIR2,* or other proapoptotic genes (*FIS1, BXI1, KEX1*; Supplementary Fig. S8), did not influence VPA sensitivity. This suggests that the growth inhibitory effects of VPA at low pH are unlikely to reflect apoptosis. Although the mechanisms underlying the effect of pH on VPA-induced biological responses remain incompletely resolved, we note that since VPA is a weak acid (pKa 4.8), neutralization of its charge at low pH may facilitate entry into yeast thereby increasing its potency. Indeed, decreasing the pH of the medium from 5 to 4 strongly increased the growth-inhibiting properties of VPA. However, formal proof that pH influences the intracellular availability of VPA in yeast awaits more detailed experiments.

Several VPA-related molecules exhibit antiepileptic but not HDAC-inhibiting properties, e.g. valpromide^3^, suggesting that VPA influences biological processes in an HDAC-independent manner. Our data support this notion since: i) VPA-induced transcriptional changes only partially overlap with those caused by deletion of HDAC encoding genes (i.e. *HDA2* and *RPD3*), or by exposure to another HDAC inhibitor, TSA, and ii) global histone acetylation patterns are not modulated in a pH-dependent manner upon exposure to VPA in budding yeast. This is consistent with studies showing very modest VPA-induced changes in H4 acetylation at K5, K8, and K12^15^ in *S. pombe*, yet in contrast to the situation in human cells where VPA causes detectable increases in global histone H3 and H4 acetylation levels^3^. Even though the basis for these inter-species differences is unknown, we speculate that VPA-induced inhibition of HDACs may only modulate chromatin structure at specific genomic loci in fungi, and that such changes may not be detectable in total histones by immunoblot.

VPA induced UPR markers under our experimental conditions. This is consistent with previous data showing that VPA causes cell wall damage and membrane/vacuolar trafficking defects in *S. pombe*^16^, which can cause UPR activation^26^,^54^. Interestingly, VPA-induced depletion of inositol also perturbs vacuolar function in budding yeast, which exacerbates the sensitivity of vacuolar mutants to VPA in medium lacking inositol^19^. However, we found that the growth-inhibiting properties of VPA are not influenced by lack of inositol in pH 4.1 SC medium (Supplementary Fig. S9), suggesting that VPA-induced inositol depletion is unlikely to account for the toxicity of this compound under our conditions. Intriguingly, mutation of the canonical UPR effectors Ire1 and Hac1 did not increase VPA sensitivity. Since Ire1/Hac1-independent transcriptional regulation of UPR genes exist in yeast^30^, it is possible that VPA-mediated modulation of such pathways contributes to the toxicity of this compound. We also note that while VPA caused modest influx of extracellular calcium, a response which has been linked to the UPR^31^, deletion of genes encoding either calcium channels, or calcineurin and calmodulin subunits, do not influence yeast proliferation in VPA. This is in sharp contrast with numerous environmental stresses, e.g. cold, iron, plant essential oils, and amiodarone, which cause fungi to rely on Cch1/Mid1-dependent signalling for survival^55^,^56^,^57^. In addition, VPA-induced transcriptional changes were found to be inconsistent with reported gene expression signatures associated with treatments known to cause elevated cytoplasmic Ca^2+^. The biological relevance of the observed calcium influx in response to VPA therefore remains unclear in *S. cerevisiae*.

We demonstrated that VPA modulates MAP kinase signalling in budding yeast. The Slt2-dependent cell wall integrity and Kss1/Fus3-dependent mating pheromone pathways are inhibited by VPA at low pH, while p38 (Hog1) phosphorylation is induced under these conditions. Since *hog1∆* cells are not sensitive to VPA, upregulation of the osmostress response pathway does not influence survival in response to this drug. On the other hand, it is unclear whether downregulation of cell wall integrity and/or mating pheromone signalling contributes to VPA-mediated growth arrest. Since VPA negatively influences cell wall integrity in fission yeast, and Slt2 promotes resistance to cell wall stress, we speculate that VPA-induced cell wall stress and concomitant inhibition of Slt2 phosphorylation may synergize to inhibit yeast proliferation. VPA-induced modulation of MAP kinase signalling has also been observed in mammalian models, but the effects (activation or inhibition) vary widely between systems. For example, in human HepG2 hepatic carcinoma cells VPA promotes MEK1/2 signalling without activating p38^58^, whereas the opposite is observed in K562 erythroid cells^59^. More studies will be needed to elucidate and compare the mechanisms underlying VPA-induced modulation of MAP kinase signalling in yeast and human cells.

VPA blocks progression in every phase of the cell cycle, leading to complete inhibition of proliferation. We found that the G1 to S transition is impeded by VPA through downregulation of Cln1/2 expression. G1 arrest has been observed in VPA-treated human glioma cells, and depends on modulation of cyclin levels^7^. Elucidation of the molecular basis of VPA-mediated reduction in Cln1/2 expression in yeast will require further experiments, although we have excluded the possible contribution of the DNA damage checkpoint kinase Mec1. Remarkably, VPA also caused rapid and reversible DNA replication arrest in yeast. We did not observe any evidence of VPA-induced DNA damage (Rad52/Rfa1 focus formation or Rad53 kinase phosphorylation) which could explain reduced progression of DNA replication forks in the presence of this drug. Our observations may prove useful toward investigating the structure of stalled DNA replication forks in the absence of DNA damage.

In conclusion, we have outlined heretofore unreported cellular effects of VPA exposure in yeast. This highlights novel research avenues aimed at understanding the molecular basis for multiple clinical and biomedical applications of this drug.

## Methods

### Yeast strains, growth conditions, chemicals

Yeast strains were generated and propagated using standard yeast genetics methods. SC: synthetic complete, YPD: yeast extract peptone dextrose. Yeast strains are described in Supplementary Methods. Valproic acid sodium salt was purchased from Sigma-Aldrich (VPA; P4543). For synchronization, Cells were arrested in G1 with 3 μg/ml α-factor for 2 hours 45 minutes. Cells were released by incubating in medium containing 50 μg/ml pronase and appropriate chemicals. For transient exposure to genotoxins, 0.033% MMS and 200 mM HU was used. After MMS, cells were washed with 2,5% sodium thiosulfate (a chemical that inactivates MMS). Nocodazole was used at a concentration of 15 μg/ml. Where applicable, appropriate dilutions of cells were plated on YPD-agar to measure viability by colony formation assays. Flow cytometry analysis is described in Supplementary Methods.

### Immunoblots

Lysates were analysed by SDS–PAGE using the following antibodies: polyclonal antibodies (AV94 and AV100) respectively against yeast histone H4 and a C-terminal peptide of H3 (a gift from Dr Alain Verreault, Université de Montréal), Monoclonal anti-H3K9ac (Cell Signaling, C5B11, Cat. No 9649), Phospho-p38 MAPK (Thr180/Tyr182) (Cell Signaling, D3F9, Cat. No 4511) and p44/42 MAPK (Thr202/Tyr204) phospho (Cell Signaling, 9101S), Histone H4K5ac (Active Motif, 39583, 39584), H4K12ac (Active motif, 39165, 39166), Anti-acetyl-histone H4K16Ac (Millipore, Cat. No 07-329), anti-acetyllysine (Immunechem, ICP0380), Anti-TAP (CBP) (Fisher, cab1001). Anti-tubulin (YOL1/34) (Abcam, ab6161), Anti-V5 (Medimab, ab27671). ECL scans of whole membranes are in Supplementary Fig. S10

### Drug susceptibility assays

For spot assays, appropriate dilutions of cultures were spotted on YPD-agar and incubated at 30 °C for 3–5 days. For growth assays in 96 well plates, cultures were diluted at 0,001 OD/ml and mixed 1:1 with medium containing drugs. Remove this sentence as the corresponding experiments are no longer presented in our study. For growth curves, saturated cultures were diluted at 0,01 OD/ml in medium +/−10 mM VPA. Optical density was monitored every 30 minutes for 48 hours. Synergy was assessed by OD determination at 630 nm after incubating cells with the two drugs in a 1:1:1 proportion. The formula


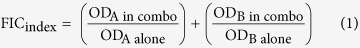


was used to calculate the Fractional Inhibitor Concentration (FIC)^41^ index as described. A FIC_index_ < 0.5 indicates synergism.

### Fluorescence microscopy

Cell samples were fixed with formaldehyde and examined using a GE DeltaVision Olympus IX71 fluorescence microscope equipped with an UIS2 60X/1,42 Plan Apo objective (Olympus), a 15-bit EDGE/sCMOS (PCO) camera, and softWoRx v.6.2.0 software. Images were analysed using Image J 1.46E. >300 cells were examined per sample.

### RNA sequencing and analysis

Details and references for comparison datasets for Venn diagrams are provided in Supplementary Methods.

Experimental details regarding *HAC1* RT-PCR, luminometry analysis of calcium intake, neutral two-dimensional gel electrophoresis, and measurement of DNA content by flow cytometry are provided as Supplementary Methods.

## Additional Information

**How to cite this article**: Desfossés-Baron, K. *et al*. Valproate inhibits MAP kinase signalling and cell cycle progression in *S. cerevisiae. Sci. Rep.* **6**, 36013; doi: 10.1038/srep36013 (2016).

**Publisher’s note:** Springer Nature remains neutral with regard to jurisdictional claims in published maps and institutional affiliations.

## Supplementary Material

Supplementary Information

Supplementary Table S1

Supplementary Table S2

## Figures and Tables

**Table 1:** Go-term analysis (biological processes) of up- and down-regulated genes in presence of VPA.

Term_ID	Description	Frequency	p-value
Up-regulated genes			
GO:0008652	cellular amino acid biosynthetic process	27/297	3.44E-10
GO:0009309	amine biosynthetic process	27/297	1.30E-09
GO:0006457	protein folding	23/297	2.73E-09
GO:0042026	protein refolding	10/297	1.75E-08
GO:0044271	cellular nitrogen compound biosynthetic process	38/297	1.53E-07
GO:0046164	alcohol catabolic process	13/297	4.01E-05
GO:0006007	glucose catabolic process	11/297	8.80E-05
GO:0009628	response to abiotic stimulus	32/297	3.02E-04
GO:0006616	SRP-dependent cotranslational protein targeting to membrane, translocation	5/297	8.13E-04
GO:0032268	regulation of cellular protein metabolic process	21/297	1.13E-03
GO:0010608	posttranscriptional regulation of gene expression	20/297	1.61E-03
GO:0009266	response to temperature stimulus	21/297	2.84E-03
GO:0015677	copper ion import	4/297	4.88E-03
GO:0006113	fermentation	5/297	5.63E-03
GO:0019748	secondary metabolic process	9/297	6.68E-03
GO:0006790	sulfur compound metabolic process	12/297	7.06E-03
**Down-regulated genes**			
GO:0022604	regulation of cell morphogenesis	9/300	9.50E-06
GO:0008643	carbohydrate transport	9/300	2.05E-05
GO:0048610	Reproductive cellular process	26/300	2.11E-04
GO:0000819	sister chromatid segregation	13/300	4.73E-04
GO:0051301	cell division	32/300	4.79E-04
GO:0006260	DNA replication	18/300	6.00E-04
GO:0007059	chromosome segregation	19/300	8.40E-04
GO:0019953	sexual reproduction	26/300	1.51E-03
GO:0000087	M phase of mitotic cell cycle	19/300	2.02E-03
GO:0000741	karyogamy	6/300	4.92E-03
GO:0007049	cell cycle	45/300	5.22E-03
GO:0071555	cell wall organization	22/300	8.5E-03
GO:0006865	amino acid transport	8/300	9.2E-03
