# Supplementary material for: Valproate inhibits MAP kinase signalling and cell cycle progression in S. cerevisiae
Source: Sci Rep. 2016 Oct 26;6:36013. doi: 10.1038/srep36013 (PMC5080547; doi:10.1038/srep36013)
Supplement: Supplementary Information [file srep36013-s1.pdf]

Valproate inhibits MAP kinase signalling and cell cycle progression in *S. cerevisiae*

Kristelle Desfossés-Baron, Ian Hammond-Martel, Antoine Simoneau
Adnane Sellam, Stephen Roberts and Hugo Wurtele

Supplementary Information

Supplementary Methods

Yeast strains: Experiments were performed using yeasts of the BY4741 genetic background except for calcium influx experiments (Fig. 3; see Luminometry analysis of calcium intake section below), microscopy experiments (Fig. 7G; W303 strains W5094-1C and W3775-12C, see below), and viability experiments (Fig. 7H; W303 strains YSC1058, U953-61A, YJT7, see below). Haploid mutant strains were from the Yeast MATa haploid deletion collection (GE Healthcare YSC1053). The wild type BY4741 strain was HWY294 (BY4743 MATa ura3Δ0 leu2Δ0 his3Δ1). The wild type W303 MATa strain was from GE Healthcare (YSC1058). TAP-tagged strains were from the Yeast TAP-tag collection (GE Healthcare YSC1177). Yeast expressing Rad53-V5-6xHis was from the Cross and Capture collection (GE Healthcare YSC5049). Other strains were described elsewhere: W5094-1C [1] (W303 MATa *ADE2 RAD5 trp1-1 LYS2 RAD52-YFP*), W3775-12C [2] (W303 MATa *RAD5 RFA1-8ALA-YFP*), HWY1634 [2] (BY4741 MATa *mec1*Δ::*HPHMX sml1*Δ::*kanMX*), U953-61A [1] (W303 MATa *trp1-1 ura3-1 his3-11,15 leu2-3,112 ade2-1 can1-100 mec1*Δ::*TRP1 sml1*Δ::*HIS3*), YJT75 [1] (W303 MATa *trp1-1 ura3-1 his3-11,15 leu2-3,112 ade2-1 can1-100 sml1*Δ::*URA3 rad53*Δ::*LEU2*), Histone point mutant-expressing strains [3] (Supplementary Fig. 1).

Measurement of DNA content by flow cytometry: Cells were fixed with 70 % ethanol prior to flow cytometry. DNA content was determined using Sytox Green (Life Technologies) as described [4]. Flow cytometry was performed on FACS Calibur instrument with the Cell Quest software. Histograms were with FlowJo 7.6.5.

Neutral two-dimensional gel electrophoresis. DNA replication intermediates were examined as described [5]. DNA was digested using the *Eco*RV and *Hind*III restriction enzymes. Southern blotting was performed using a probe specific to the *ARS305* replication origin [1].

Luminometry analysis of calcium intake: The *cch1Δ* mutant was derived from JK9-3da (MATa *leu2-3, 112Δ, his4Δ trp1Δ ura3-52Δ*) by replacing *CCH1* by KanMX. Yeasts were transformed with pEVP11/AEQ (a plasmid bearing Apoaequorin gene and *LEU2*, provided by Dr Patrick Masson, University of Wisconsin-Madison) [6]. 2 mM EGTA and 8 mM VPA was added to SC-Leu using stock solutions of EGTA (50 mM Na_2EGTA, 10 mM HEPES, 2 % glucose, pH 7,0 with NaOH) and 200 mM VPA respectively. Exponentially growing cells were resuspended in SC-Leu to an OD_{600} of 2,4. 4 µl of 0,5 mM coelentrazine (Prolume, USA) in methanol was added to 1 ml of cells and incubated in the dark for 2 hours at 30°C. Luminescence from 20 µl samples of cells (OD_{600} of 8) was recorded as reported [7]. VPA was added after 40 seconds in 200 µl of SC-Leu, SC-Leu supplemented with 2 mM EGTA or BAPTA buffer (10 mM BAPTA, 50 mM MES, 2% glucose, pH 5,5). Luminescence (in arbitrary units (AU) per second) was measured for at least 400 seconds after which cells were lysed with 1,6 M $CaCl_2$ in 20% (v/v) ethanol to determine summed luminescence. Total luminescence was in significant excess over luminescence induced by VPA indicating that availability of aequorin-coelentrazine complex was not limiting.

***HAC1* RT-PCR**: Total RNA was extracted from 50 OD of cells using standard hot phenol procedures. For *HAC1* RT-PCR, cDNA were prepared using M-MLV reverse transcriptase (Thermo-Fisher cat. #28025-013) and RNase OUT Recombinant Ribonuclease Inhibitor (Thermo-Fisher cat. #10777-019) following manufacturer's protocols. PCR primers were as described [8].

RNA sequencing and analysis: 4 ug of total RNA was used for RNA sequencing; samples were analysed in triplicate for all conditions. Quality of total RNA was assessed with a BioAnalyzer Nano (Agilent). The KAPA mRNAseq stranded kit (KAPA, Cat no. KK8420) was used to make librairies. Ligation was done with 100 nM final concentration of Illumina index; 6 PCR cycles were required to amplify cDNA libraries. cDNA libraries were quantified using QuBit and BioAnalyzer. Librairies were diluted to 10 nM and normalized by qPCR using the KAPA quantification kit (KAPA; Cat no. KK4973). Libraries were pooled to equimolar concentration. Sequencing was performed using a Illumina Miseq using the Miseq Reageant Kit v2 (50 cycle single-read) and 7 pM of the pooled library. Cluster density was targeted at around 800k clusters/mm^2. Approx. 3-6 million reads were generated by samples. Library preparation and sequencing was performed at the Institute for Research in Immunology and Cancer's Genomics Platform (Université de Montreal).

Sequences were trimmed for sequencing adapters and aligned to the *Saccharomyces cerevisiae* reference genome using Tophat version 2.0.10. The UCSC sacCer3 genome was used along with July 2015 gene annotations. Gene expression was quantified using

Cufflinks-2.1.1 and htseq-count (with intersection-strict argument). Differential expression was assessed using DESeq2 version 1.6.2 in which readcounts were normalized and grouped by condition. See Supplementary table S1 for RNA profiling data [9]. Venn diagrams were made using the http://www.bioinformatics.lu/venn.php web server. The top 300 up- or down-regulated genes were compared using Venn diagrams across datasets. Comparison transcriptional profiling datasets were: Trichostatin A (TSA),[10] sin3Δ and hda1Δ mutants[10], DTT [11], heat shock [12], tunicamycin [13], medium alkalinisation[14], elevated extracellular $CaCl_2$ [15], zymolyase-mediated cell wall digestion [16], diploid-specific genes [17]. The statistical significance of the overlaps was calculated based on the hypergeometric distribution as previously described [18]. Briefly, the formula:

$$p = \sum_{i=k}^{\min(mn)} \binom{m}{i}\binom{N-m}{n-i} / \binom{N}{n}$$

Equation S1

was used to calculate the enrichment p-value (p) with the R v.3.2.5 software. The p-value indicates the probability, by random chance, of having k or more common genes when randomly picking m and n genes from a pool of N total genes. We used the smallest number of probed genes from each experiment as the total number of genes (N). GO-Terms analyses were performed using VPA-modulated genes (297 up- and 300 down-regulated genes) with DAVID version 6.7 [19]. GO-Terms with a p-value of less than 0.01 were then summarized with the REViGO software[20] to generate the data presented in Table 1.

References

1. Wurtele, H. *et al.* Histone h3 lysine 56 acetylation and the response to DNA replication fork damage. *Mol. Cell. Biol.* **32,** 154–172 (2012).
2. Bélanger, F. *et al.* Mutations in Replicative Stress Response Pathways Are Associated with S Phase-Specific Defects in Nucleotide Excision Repair. *J. Biol. Chem.* (2015). doi:10.1074/jbc.M115.685883
3. Nakanishi, S. *et al.* A comprehensive library of histone mutants identifies nucleosomal residues required for H3K4 methylation. *Nat. Struct. Mol. Biol.* **15,** 881–888 (2008).
4. Haase, S. B. & Reed, S. I. Improved flow cytometric analysis of the budding yeast cell cycle. *Cell Cycle Georget. Tex* **1,** 132–136 (2002).
5. Liberi, G. *et al.* Methods to study replication fork collapse in budding yeast. *Methods Enzymol.* **409,** 442–462 (2006).
6. Batiza, A. F., Schulz, T. & Masson, P. H. Yeast respond to hypotonic shock with a calcium pulse. *J. Biol. Chem.* **271,** 23357–23362 (1996).
7. Roberts, S. K., McAinsh, M. & Widdicks, L. Cch1p mediates Ca2+ influx to protect Saccharomyces cerevisiae against eugenol toxicity. *PloS One* **7,** e43989 (2012).
8. Mori, T. *et al.* Dual Functions of Yeast tRNA Ligase in the Unfolded Protein Response: Unconventional Cytoplasmic Splicing of HAC1 Pre-mRNA Is Not Sufficient to Release Translational Attenuation. *Mol. Biol. Cell* **21,** 3722–3734 (2010).
9. Love, M. I., Huber, W. & Anders, S. Moderated estimation of fold change and dispersion for RNA-seq data with DESeq2. *Genome Biol.* **15,** 550 (2014).
10. Bernstein, B. E., Tong, J. K. & Schreiber, S. L. Genomewide studies of histone deacetylase function in yeast. *Proc. Natl. Acad. Sci. U. S. A.* **97,** 13708–13713 (2000).

11. Leber, J. H., Bernales, S. & Walter, P. IRE1-independent gain control of the unfolded protein response. *PLoS Biol.* **2,** E235 (2004).

12. Causton, H. C. *et al.* Remodeling of yeast genome expression in response to environmental changes. *Mol. Biol. Cell* **12,** 323–337 (2001).

13. Travers, K. J. *et al.* Functional and Genomic Analyses Reveal an Essential Coordination between the Unfolded Protein Response and ER-Associated Degradation. *Cell* **101,** 249–258 (2000).

14. Casamayor, A. *et al.* The role of the Snf1 kinase in the adaptive response of Saccharomyces cerevisiae to alkaline pH stress. *Biochem. J.* **444,** 39–49 (2012).

15. Yoshimoto, H. *et al.* Genome-wide analysis of gene expression regulated by the calcineurin/Crz1p signaling pathway in Saccharomyces cerevisiae. *J. Biol. Chem.* **277,** 31079–31088 (2002).

16. García, R., Rodríguez-Peña, J. M., Bermejo, C., Nombela, C. & Arroyo, J. The High Osmotic Response and Cell Wall Integrity Pathways Cooperate to Regulate Transcriptional Responses to Zymolyase-induced Cell Wall Stress in Saccharomyces cerevisiae. *J. Biol. Chem.* **284,** 10901–10911 (2009).

17. Galitski, T., Saldanha, A. J., Styles, C. A., Lander, E. S. & Fink, G. R. Ploidy Regulation of Gene Expression. *Science* **285,** 251–254 (1999).

18. Zhou, Q., Chipperfield, H., Melton, D. A. & Wong, W. H. A gene regulatory network in mouse embryonic stem cells. *Proc. Natl. Acad. Sci.* **104,** 16438–16443 (2007).

19. Huang, D. W. *et al.* The DAVID Gene Functional Classification Tool: a novel biological module-centric algorithm to functionally analyze large gene lists. *Genome Biol.* **8,** R183 (2007).

20. Supek, F., Bošnjak, M., Škunca, N. & Šmuc, T. REVIGO Summarizes and Visualizes Long Lists of Gene Ontology Terms. *PLoS ONE* **6,** e21800 (2011).

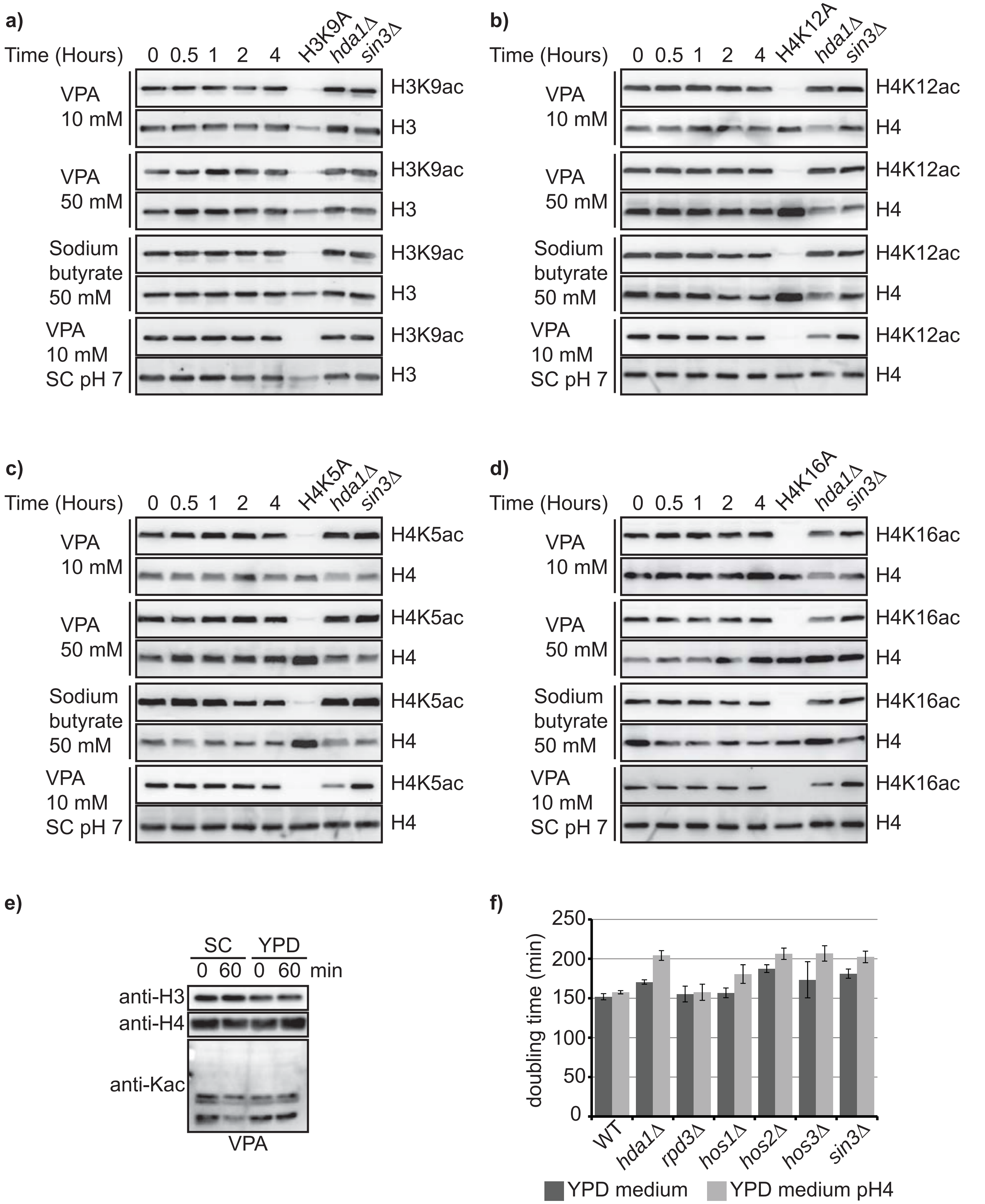

Supplementary Fig. S1: Environmental pH does not significantly modulate the acetylation of Sin3/Rpd3 and Hda1 chromatin targets upon VPA exposure. A-D) pH does not influence H3K9, H4H5, H4K12 or H4K16 acetylation levels upon VPA exposure. Cells were treated with VPA or sodium butyrate at the indicated concentration and pH. Sample were processed for immunoblot analyses with the appropriate antibodies at indicated time points. Yeast strains expressing non-acetylatable lysine to alanine mutant histone alleles were used as antibody specificity controls. E) As in A, except with an antibody against acetylated lysine residues. F) HDAC mutants do not present growth defects in acidic medium. Cells were diluted at 0,005 OD/mL and incubated in the indicated medium for 48 h. Doubling times were calculated from OD630 mesurements every 30 minutes for 48h.

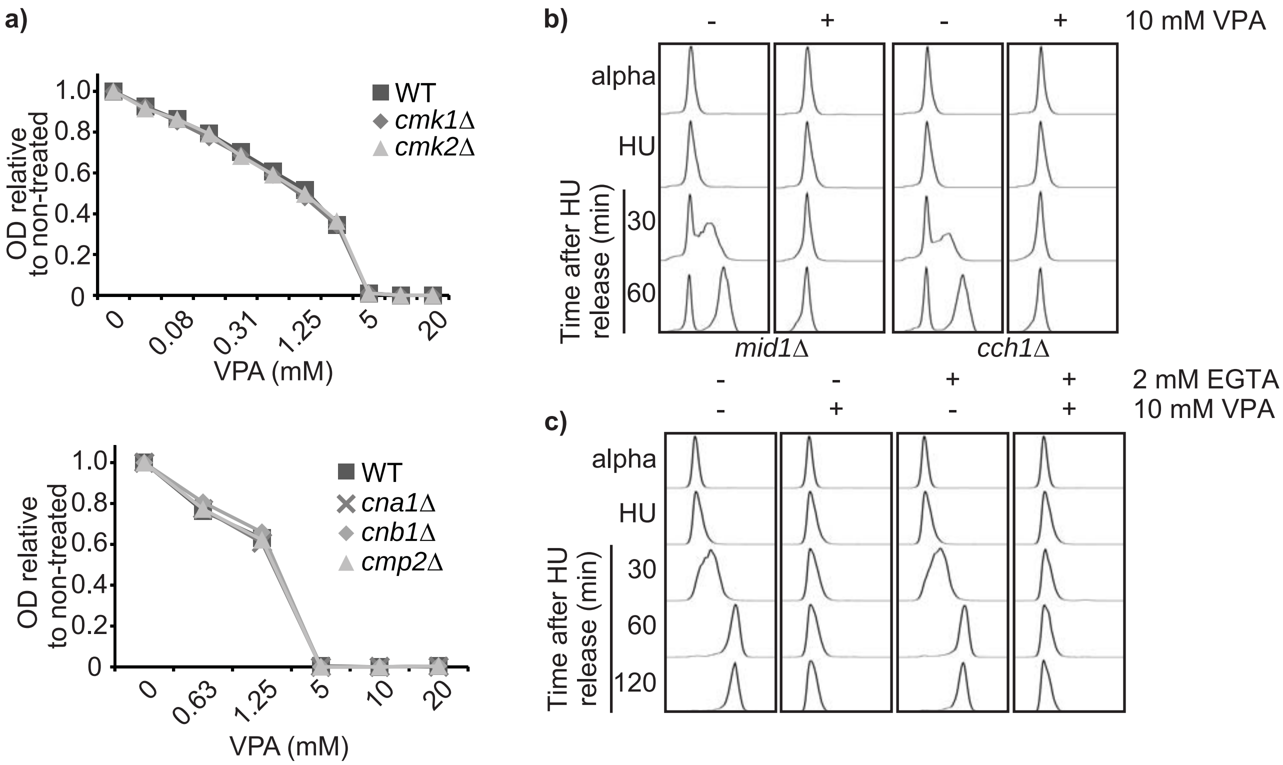

Supplementary Fig. S2: VPA-induced growth inhibition is not influenced by calcium signalling. A) Calcineurin or calmodulin mutants are not hypersensitive to VPA. Cell growth was monitored by OD_{630} measurements after 48h. B) Cell cycle arrest induced by VPA does not require the Cch1-Mid1 calcium channel. Cells were synchronized in G1 and released in S phase with 200 mM HU for 90 minutes. Cells were then incubated in SC +/- 10 mM VPA for 60 minutes. Sample were processed for DNA content analysis by flow cytometry. C) VPA-induced inhibition of S phase progression does not depend on extracellular calcium. As in B, except that cells were incubated after HU in medium +/- 10 mM VPA and/or +/- 2 mM EGTA.

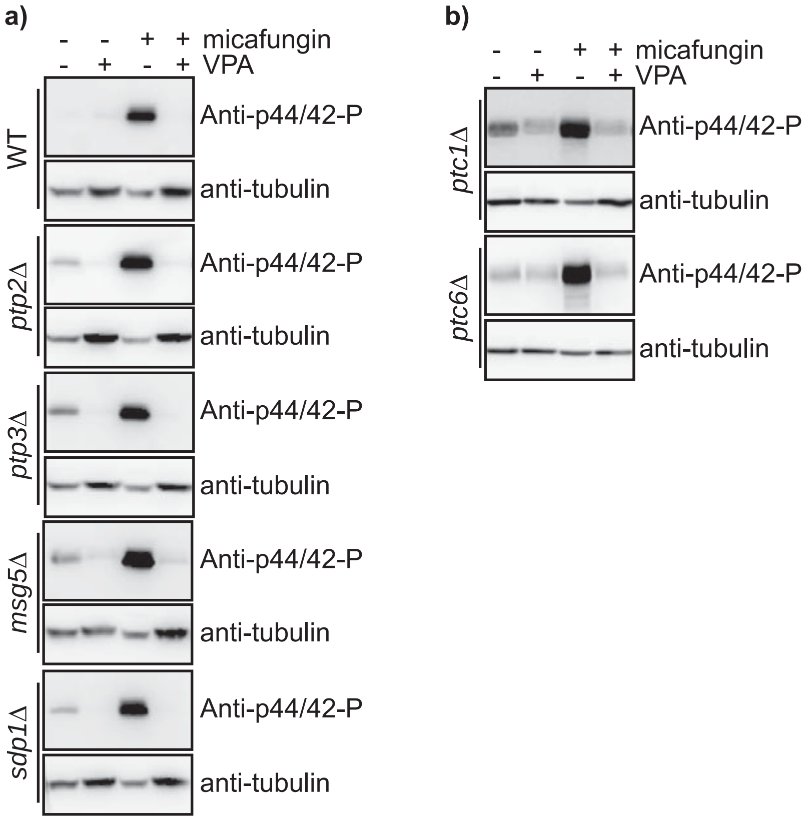

Supplementary Fig. S3: Deletion of phosphatases do not prevent VPA-induced inhibition of Slt2 phosphorylation. A) Lack of Slt2 phosphatases does not affect the inhibition of Slt2 phosphorylation by VPA. Cells were incubated in SC +/- 10 mM VPA and +/- 2 mg/mL micafungin for 60 minutes. Sample were processed for immunoblot with the indicated antibodies. B) Lack of Mkk1/2-phosphatases does not affect the inhibition of Slt2 phosphorylation by VPA. Cells were treated as in A.

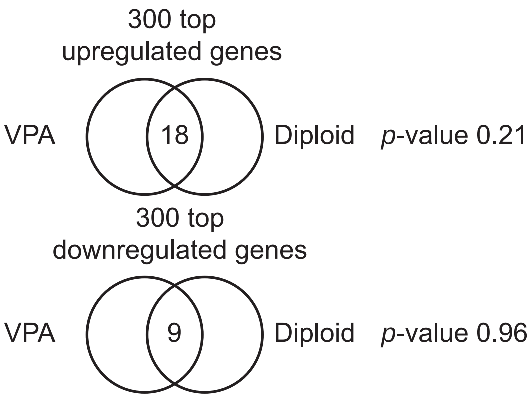

Supplementary Fig. S4: Diploid-specific vs VPA-induced genes. The top 300 up- and downregulated genes in response to VPA were compared to published datasets using Venn diagrams; p-values were calculated as described in Methods.

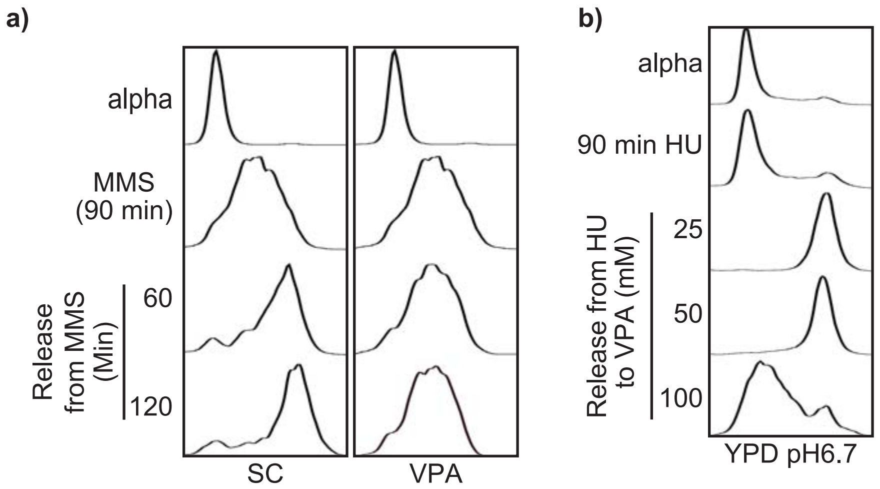

Supplementary Fig. S5: VPA inhibits S phase progression. A) VPA inhibits resumption of DNA replication after MMS exposure. Cells were synchronized in G1 and released toward S in the presence of 0,033 % MMS for 90 minutes. Cells were then incubated in SC +/- 10 mM VPA for 120 minutes. Sample were processed for DNA content analysis by flow cytometry. B) High concentrations of VPA inhibit resumption of DNA replication after HU exposure in pH 6.7 YPD. Cells were synchronized in G1 and released in S phase in the presence of 200 mM HU for 90 minutes. Cells were then incubated in SC containing the indicated concentration of VPA for 60 minutes. Samples were processed for DNA content analysis by flow cytometry.

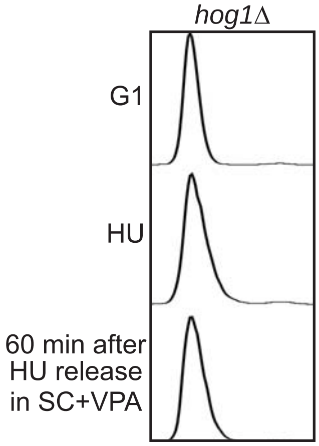

Supplementary Fig S6: VPA-mediated inhibition of DNA replication does not depend on Hog1. Cells were synchronized in G1 and released toward S phase in SC medium containing 200 mM HU for 60 minutes. Cells were washed and incubated with SC +/- 10 mM VPA. Samples were processed for DNA content analysis by flow cytometry.

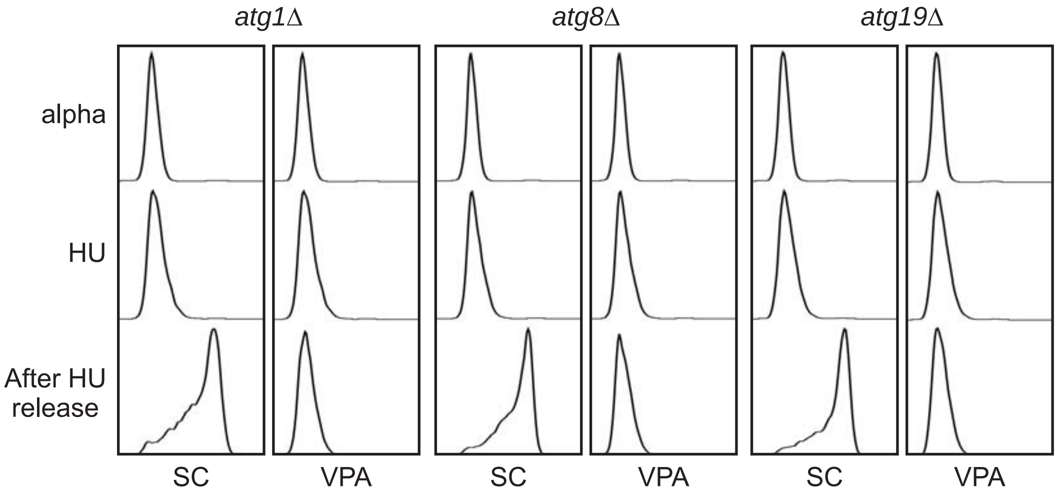

Supplementary Fig. S7: VPA-mediated inhibition of DNA replication does not require genes of the autophagy pathway. Cells were synchronized in G1 and released toward S phase in SC medium containing 200 mM HU for 60 minutes. Cells were washed and incubated with SC +/- 10 mM VPA for 60 minutes. Samples were then processed for DNA content analysis by flow cytometry.

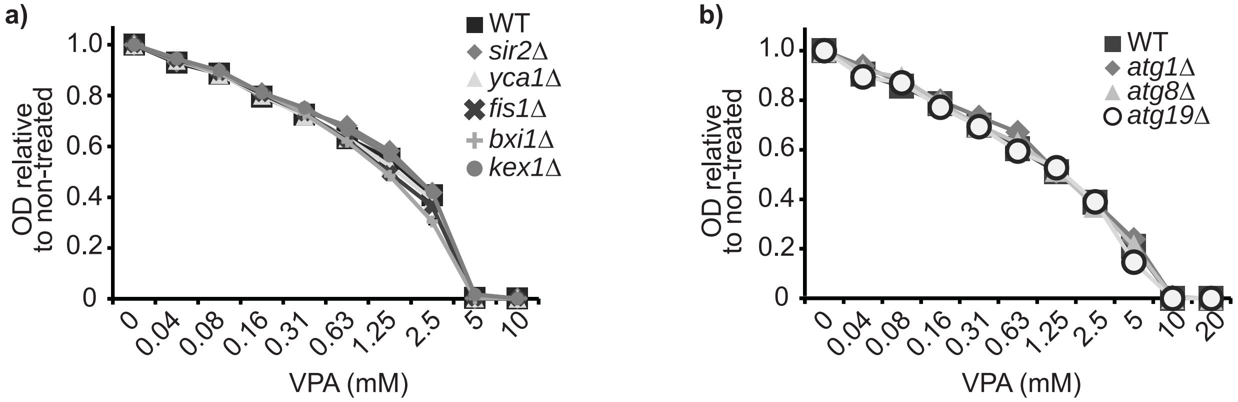

Supplementary Fig. S8: Apoptosis and autophagy genes do not influence VPA sensitivity at low pH. A) Apoptosis mutants are not resistant to VPA in SC medium. Cell growth was monitored by OD_{630} mesurements after 48h. B) Autophagy mutants are not resistant to VPA in SC medium. Cells were treated as in A.

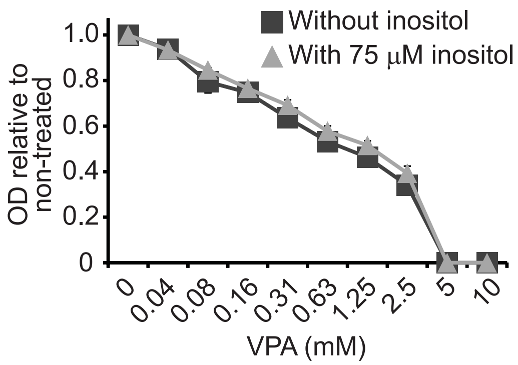

Supplementary Fig. S9: Inositol concentration in SC medium does not modulate VPA sensitivity.
Cell growth was monitored by OD_{630} mesurements after 48h.

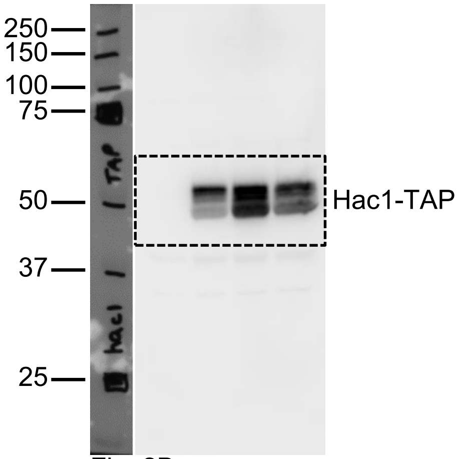

Fig. 2B

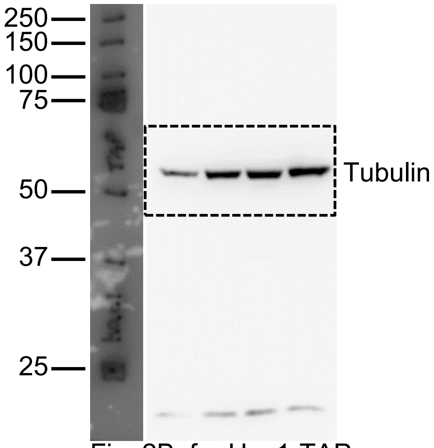

Fig. 2B; for Hac1-TAP

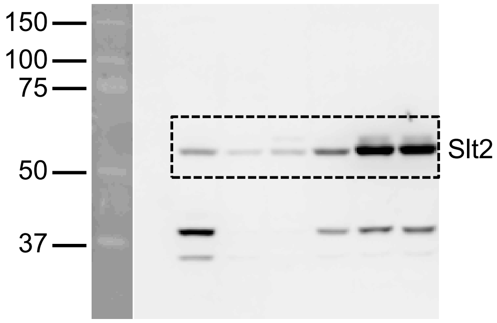

Fig. 4B Top panel

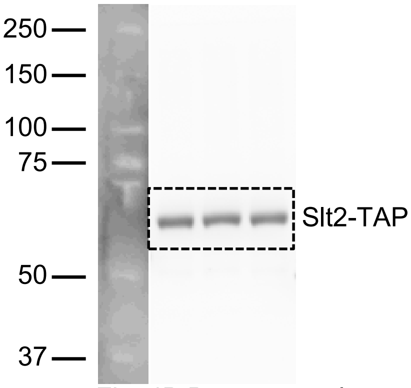

Fig. 4B Bottom panel

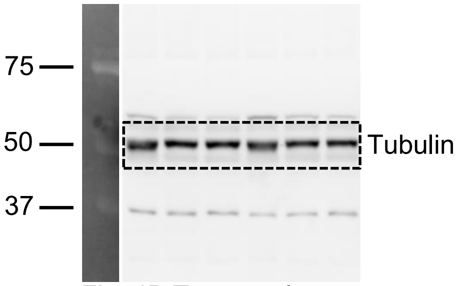

Fig. 4B Top panel

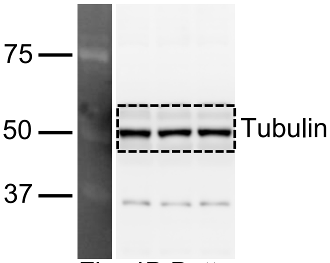

Fig. 4B Bottom

Supplementary Fig. S10 (continued below; legend on last page)

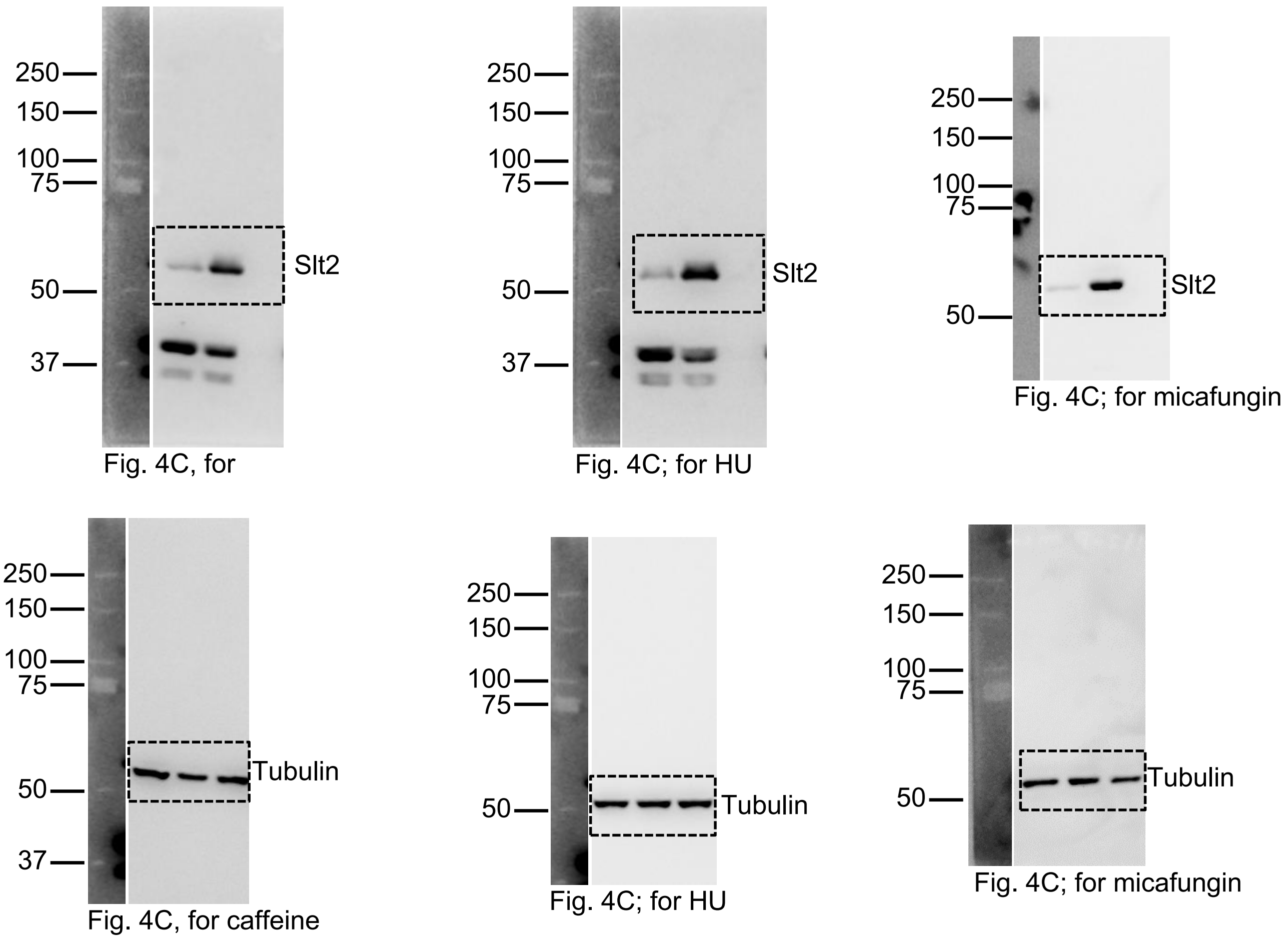

Supplementary Fig.S10 (continued below; legend on last page)

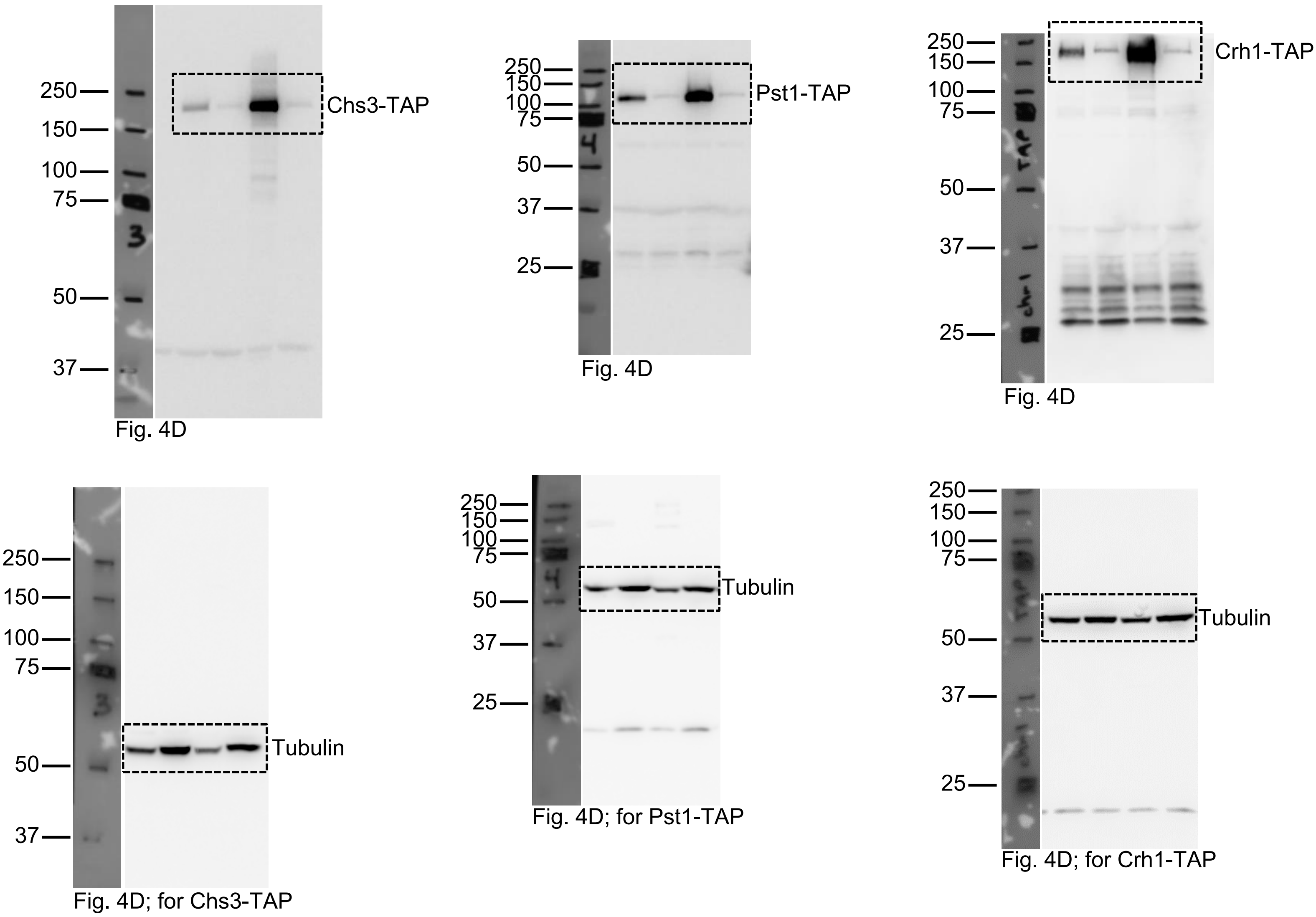

Supplementary Fig. S10 (continued below; legend on last page)

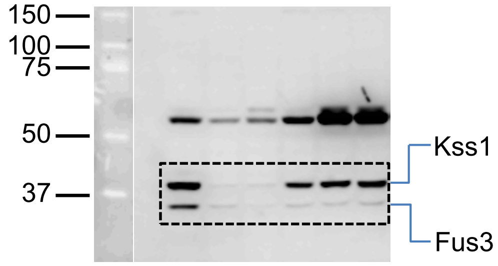

Fig. 5A

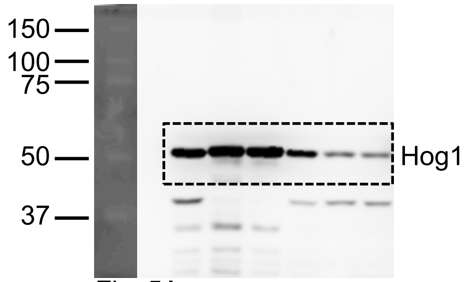

Fig. 5A

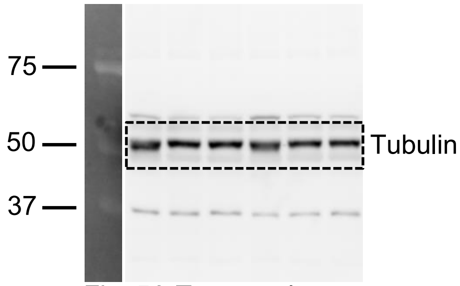

Fig. 5A Top panel
Same membrane than 4b

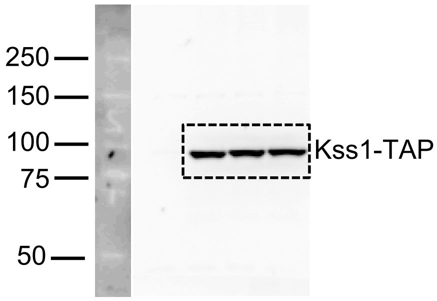

Fig. 5B

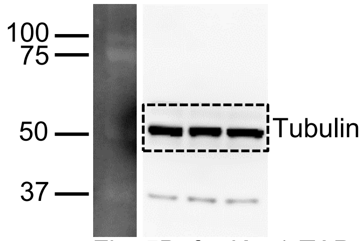

Fig. 5B; for Kss1-TAP

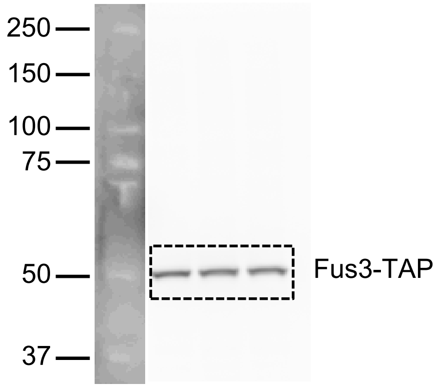

Fig. 5B

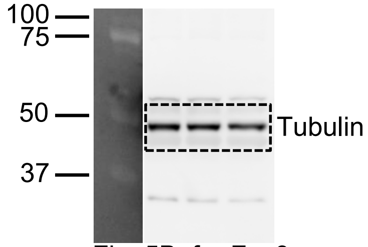

Fig. 5B; for Fus3-

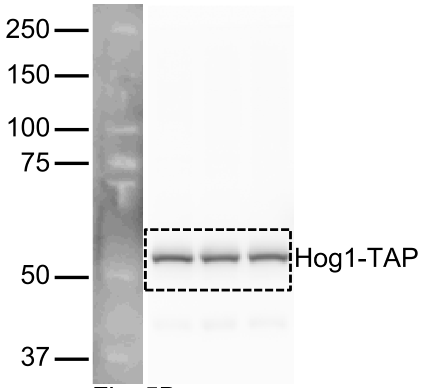

Fig. 5B

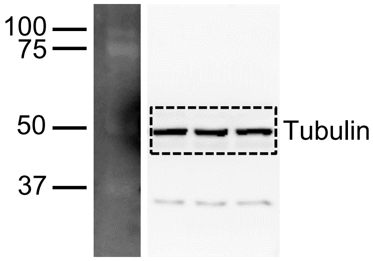

Fig. 5B; for Hog1-TAP

Supplementary Fig. S10 (continued below; legend on last page)

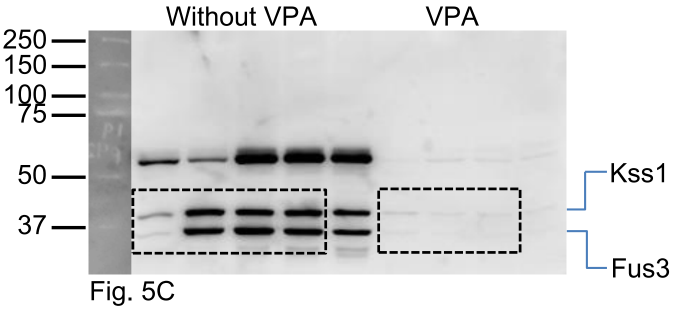

Fig. 5C

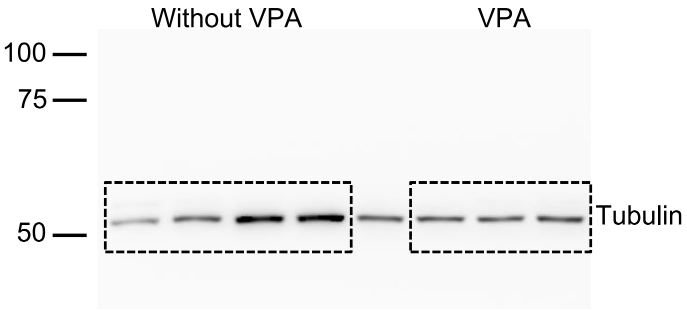

Fig. 5C

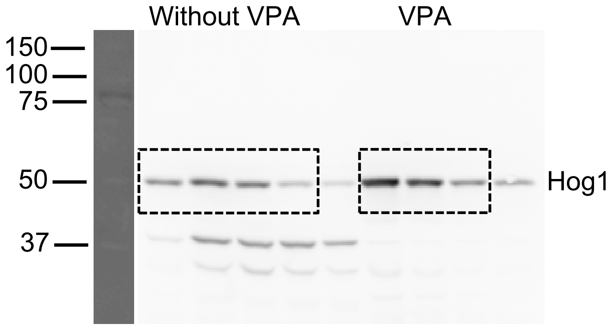

Fig. 5C

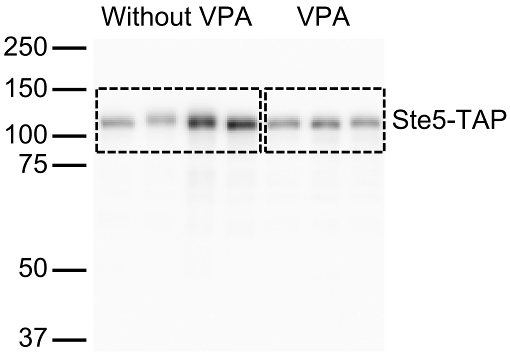

Fig. 5C

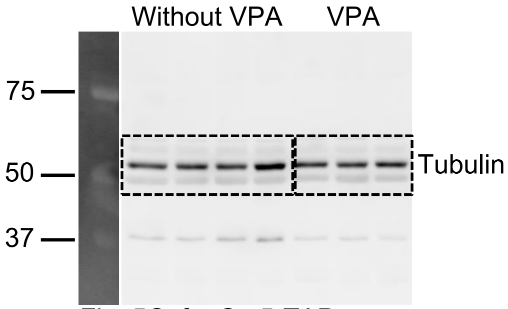

Fig. 5C; for Ste5-TAP

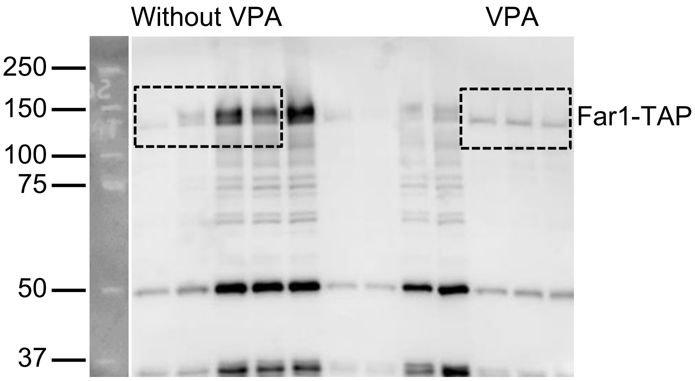

Fig. 5C

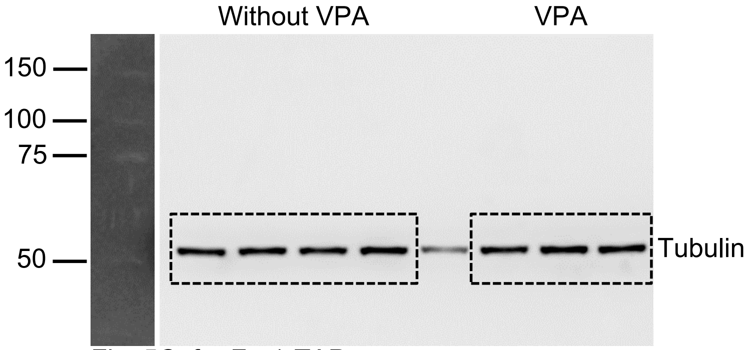

Fig. 5C; for Far1-TAP

Supplementary Fig. S10 (continued below; legend on last page)

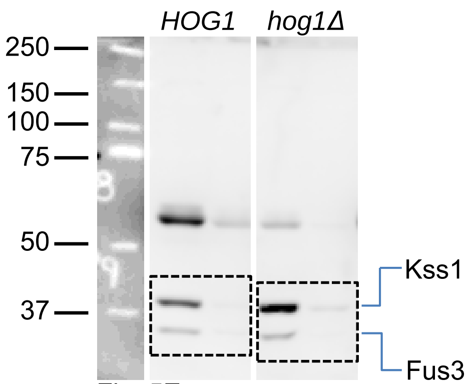

Fig. 5E

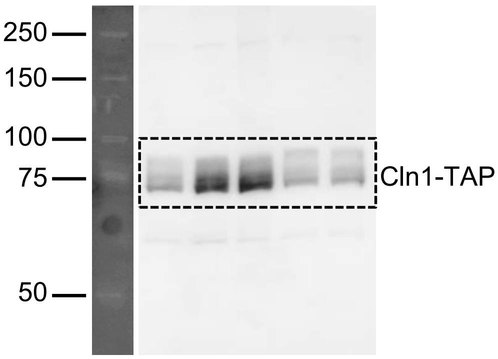

Fig. 6C

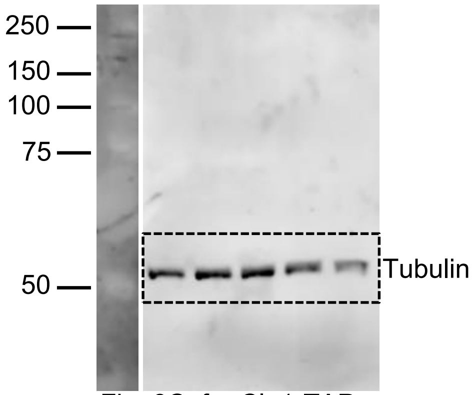

Fig. 6C; for Cln1-TAP

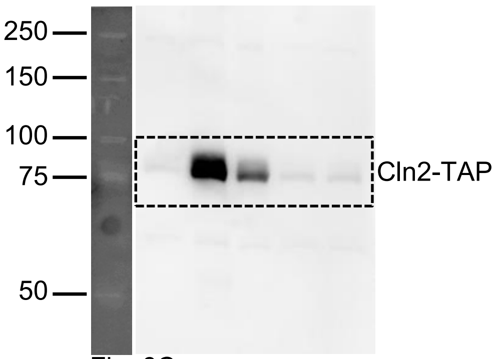

Fig. 6C

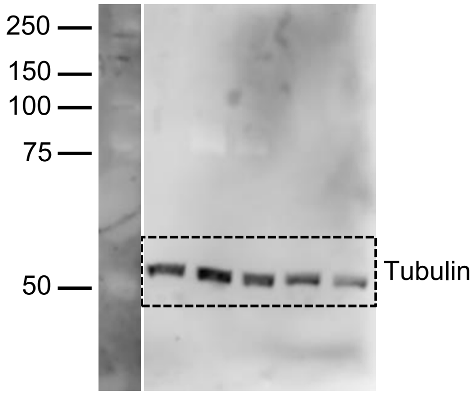

Fig. 6C; for Cln2-TAP

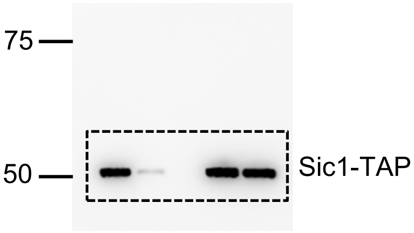

Fig. 6C

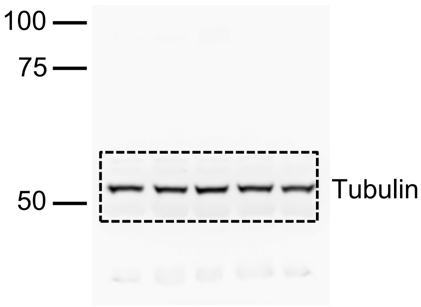

Fig. 6C; for Sic1-TAP

Supplementary Fig. S10 (continued below; legend on last page)

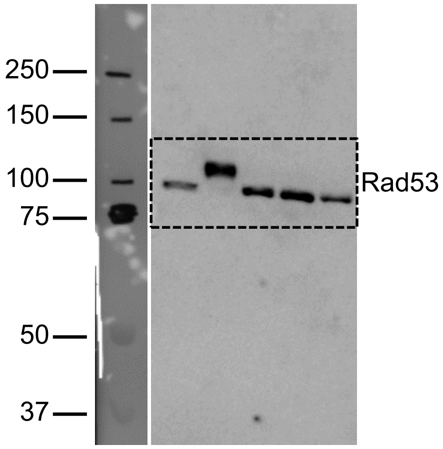

Fig. 8I; top panel

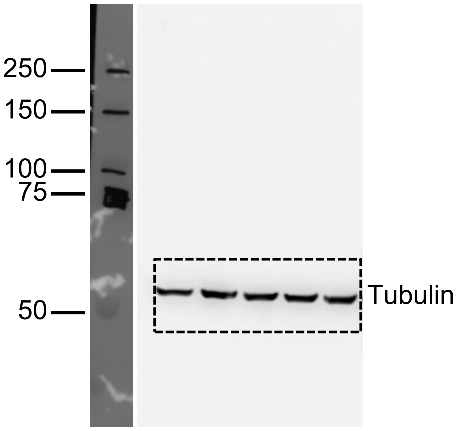

Fig. 8I; top panel

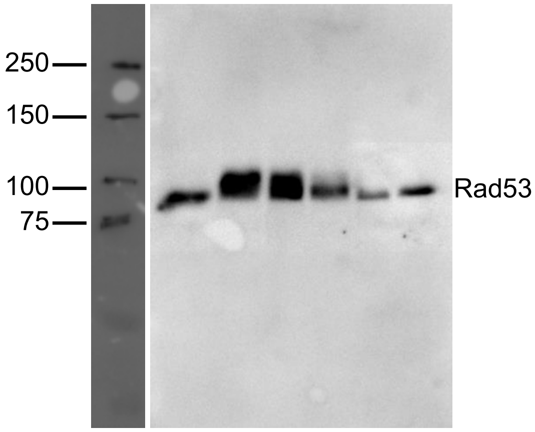

Fig. 8I; bottom panel; for SC

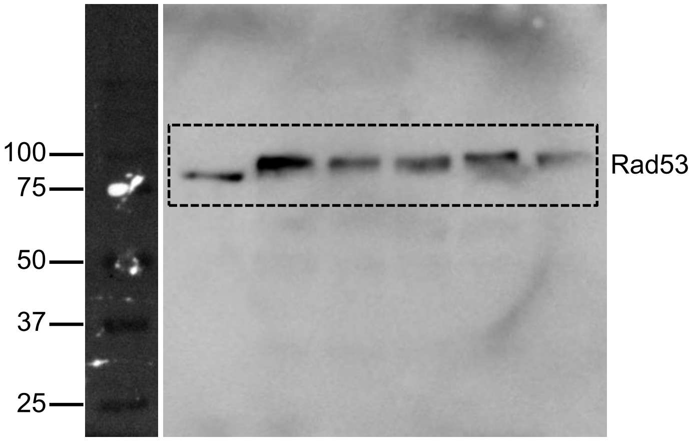

Fig. 8I; bottom panel; for HU

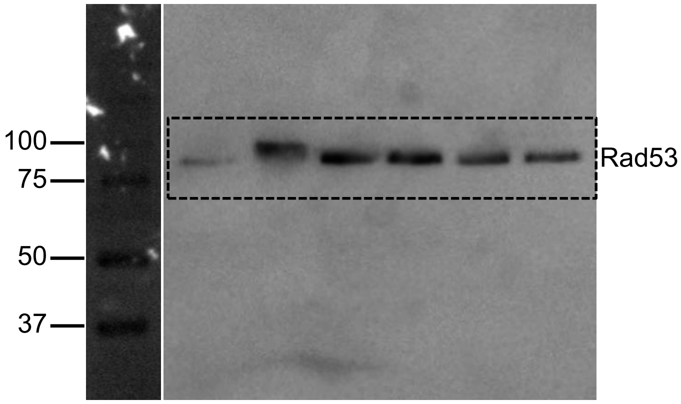

Fig. 8I; bottom panel; for VPA

Supplementary Fig. S10 (continued below; legend on last page)

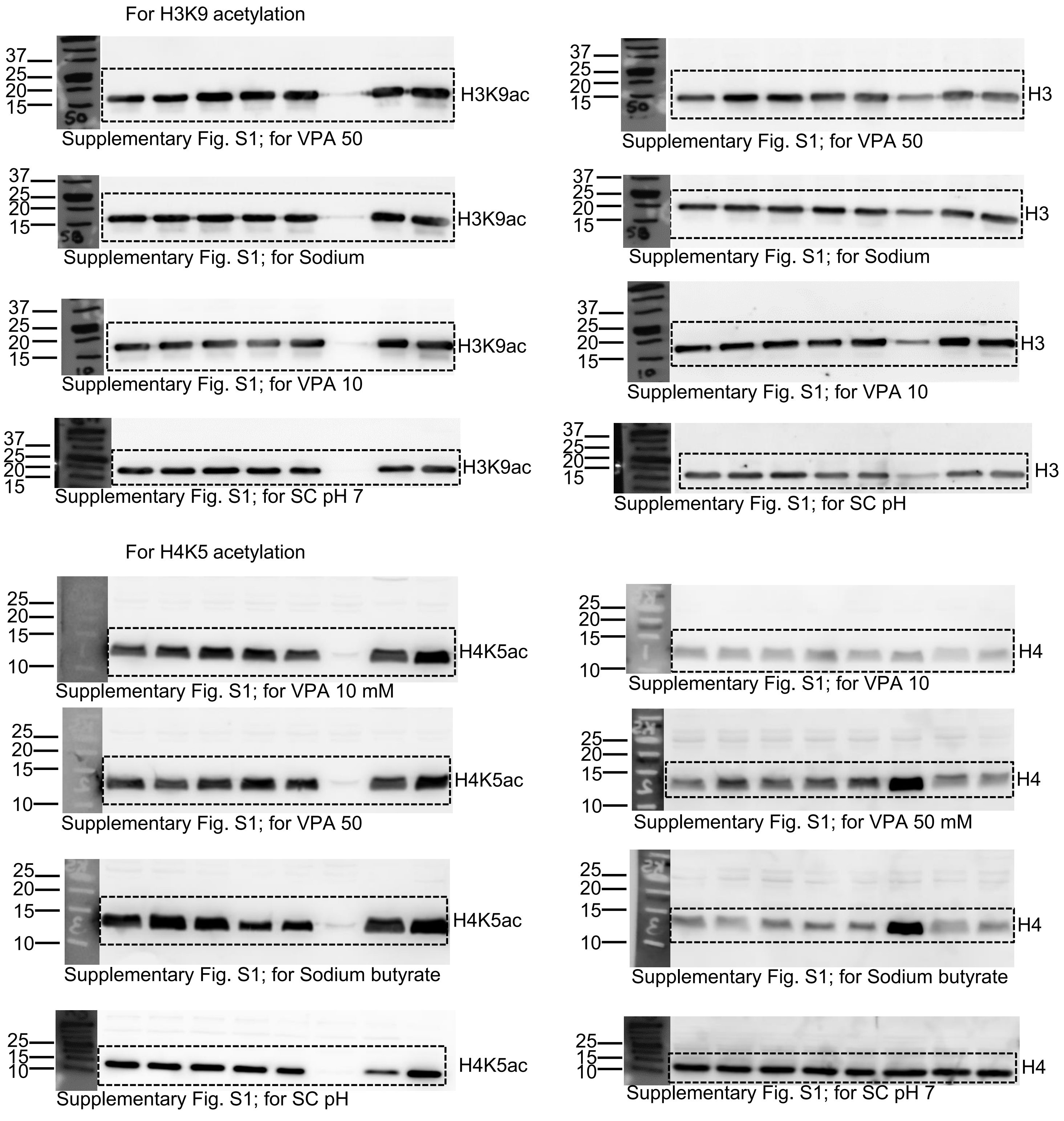

Supplementary Fig. S10 (continued below; legend on last page)

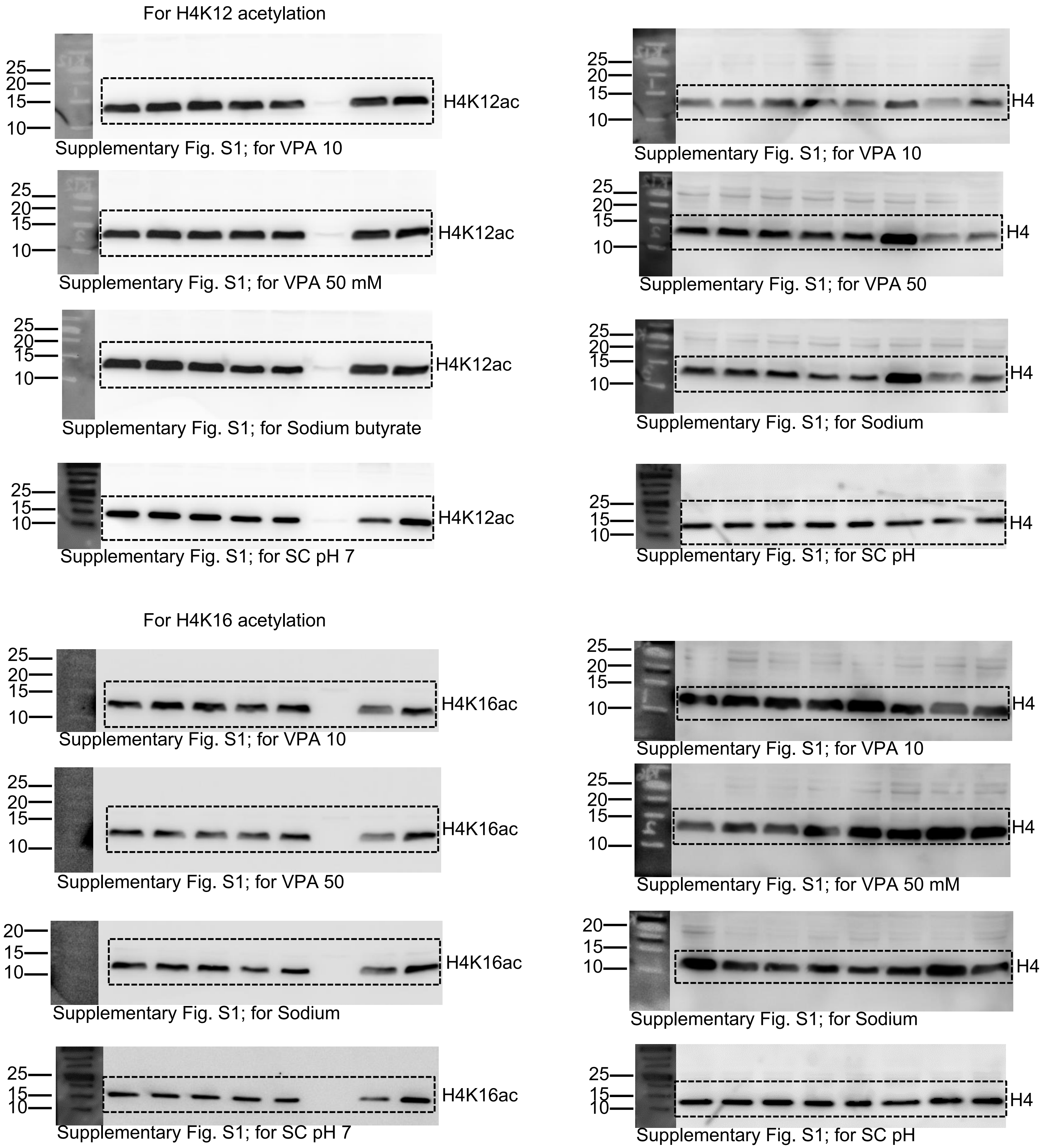

Supplementary Fig. S10 (continued below; legend on last page)

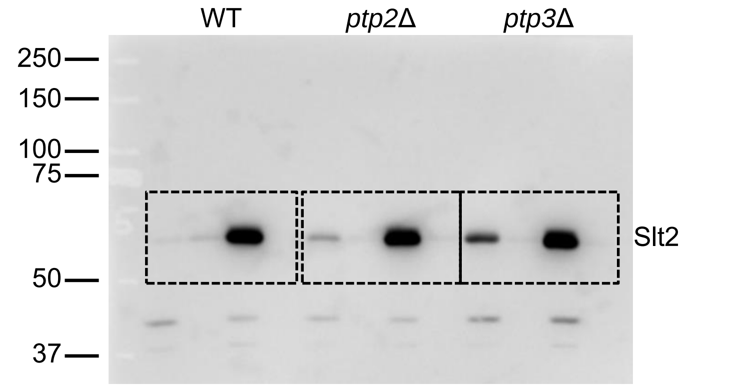

Supplementary Fig. S3

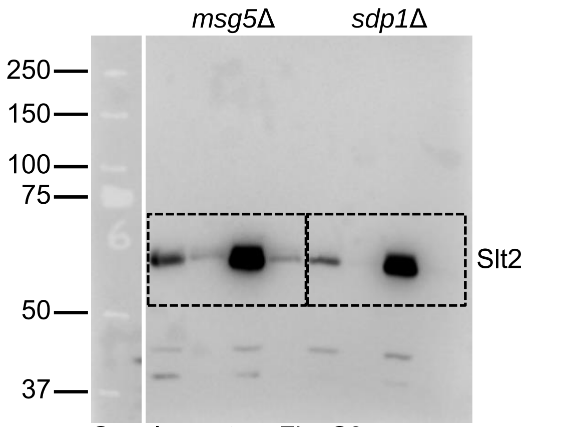

Supplementary Fig. S3

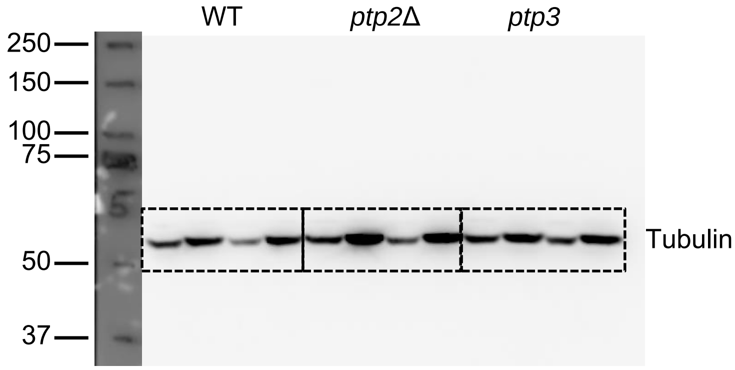

Supplementary Fig. S3

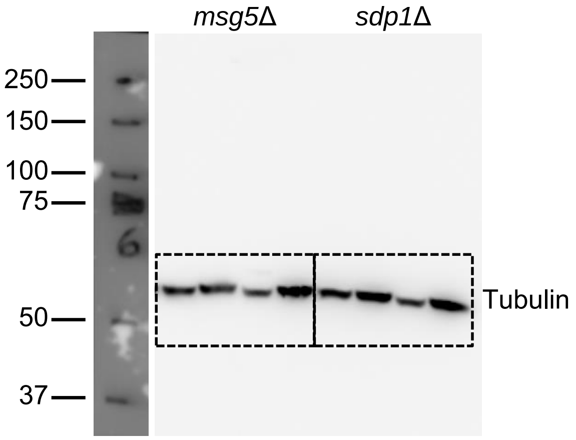

Supplementary Fig. S3

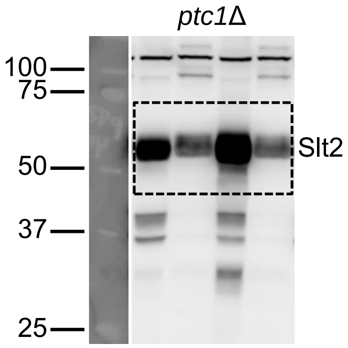

Supplementary Fig. S3

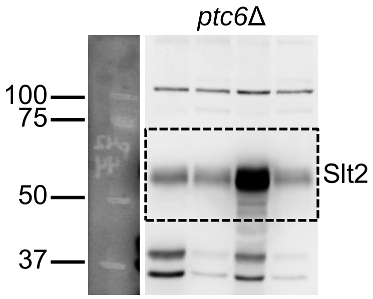

Supplementary Fig. S3

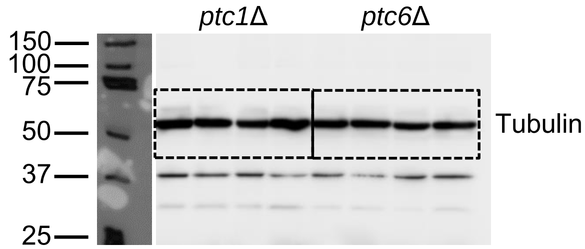

Supplementary Fig. S3

Supplementary Fig. S10 Scans of the uncropped images of acrylamide gels that were used in the manuscript. Boxes indicate approximate portions of the gels that are included in the figures. The corresponding figure is indicated under each scan.

Supplementary Table S3 : UPR genes GO-term analysis

GO term category	Genes
**Vesicle-mediated transport**	*RIM8, SSA1, SPO20, BTN2, EGD2*
**Proteolytic degradation**	*RPN4, RPN1, UBC4, KAR2*
**Protein folding**	*SIS1, YDJ1, MDJ1, FES1, SSB1, SSA2, HSP104, SSA1, SSA4, APJ1, HSP82, HSP26, HSP78, SSE1, SSE2, HSC82, ZUO1, ERO1, CPR6, CUR1*
**Amino acid metabolism**	*ASN1, LYS20, BNA3, STR3, SER2, HIS5, MET16, IRC7, AAT1, SAM1, SAH1, GLT1, ILV3, CPA2, ADE4, ILV2, SER3, MET22, SAM2, CYS3, GLN1, CIT2, HIS1, MCM1, HIS4, HOM3, MET14, ARG4, BAT1, MUP1, MUP3, ARO3, ARO4*
**Lipid metabolism and metabolism**	*INO1, OPI10, OPI3, PDR16, GRE2, EEB1, EHT1, ICT1, OYE2, OYE3, CIT3, MUM3, PDR12, SNQ2*
**Cellular transport**	*TPO1, TPO2, TPO4, AQY2, FET4, JEN1, MPC2, PIC2, VMA9, YMC2, ZRT1, HXK2, HXT11, HXT9, CTR1, FRE7, FRE1, OAC1, FIT2, PUG1*
**Cell wall proteins and biogenesis**	*TIR2, SPI1, YGP1, OSW1, SPS1*

Supplementary Table S4: VPA-induced change in expression of genes involved in the pheromone and cell wall integrity pathways

**Pheromone pathway**			
**Systematic name**	**Standard name[a]**	**Function**	**Log2 Fold Change[b]**
YFL026W	Ste2	Receptor for alpha-factor pheromone	-0.207
YHR005C	Gpa1	Alpha subunit of the G protein	-0.623
YJR086W	Ste18	Gamma subunit of the G protein	0.239
YOR212W	Ste4	Beta subunit of the G protein	0.083
YDR103W	Ste5	Scaffold protein	-0.201
YHL007C	Ste20	Cdc42p-activated signal transducing kinase; activate Ste11	-0.880
YLR362W	Ste11	MEK kinase	-0.055
YCL032W	Ste50	Binds to Ste11 and aids and/or helps maintain in its activation	-0.222
YDL159W	Ste7	MAPKK	-0.060
YGR040W	Kss1	MAP kinase	-0.494
YBL016W	Fus3	MAP kinase	-0.594
YJL157C	Far1	CDK inhibitor and nuclear anchor	-1.141
YPL049C	Dig1	MAPK inhibitor of Ste12	0.616
YDR480W	Dig2	MAPK inhibitor of Ste12	-0.112
YHR084W	Ste12	Transcription factor	-0.240
**Cell wall integrity pathway**			
**Systematic name**	**Standard name[a]**	**Function**	**Log2 Fold Change[b]**
YOR008C	Wsc1	Sensor-transducer of the stress-activated PKC1-MPK1 signaling pathway	0.167
YNL283C	Wsc2	Sensor-transducer of the stress-activated PKC1-MPK1 signaling pathway	-0.975
YOL105C	Wsc3	Sensor-transducer of the stress-activated PKC1-MPK1 signaling pathway	-0.642
YGR023W	Mtl1	Plasma membrane sensor; involved in cell wall integrity signaling	0.743
YLR332W	Mid2	O-glycosylated plasma membrane protein; acts as a sensor for cell wall integrity pathway	0.255
YGR070W	Rom1	Rho1-Guanine nucleotide exchange factor (GEF)	-1.288
YLR371W	Rom2	Rho1-Guanine nucleotide exchange factor (GEF)	-0.462
YLR425W	Tus1	Rho1-Guanine nucleotide exchange factor (GEF)	-0.299
YPR165W	Rho1	GTP-binding protein of the rho subfamily of Ras-like proteins	0.331
YBL105C	Pkc1	Protein serine/threonine kinase; activates Bck1p	-0.607
YJL095W	Bck1	MAPKKK; activated by Pck1p; activates Mkk1/2p	-0.916
YOR231W	Mkk1	MAPKK; activated by Bck1p; target Slt2p	0.329
YPL140C	Mkk2	MAPKK; activated by Bck1p; target Slt2p	0.010
YHR030C	Slt2	MAP kinase; involved in cell wall integrity and cell cycle progression	-0.449
YPL089C	Rlm1	MADS-box transcription factor; phosphorylated and activated by Slt2p	0.034
YOR134W	Bag7	Rho1-Guanine nucleotide exchange factor (GEF)	-0.316
YDR389W	Sac7	Rho1-GTPase activating protein (GAP)	-0.244

YER155C	Bem2	Rho1-GTPase activating protein (GAP)	-0.944
YDL240W	Lrg1	Rho1-GTPase activating protein (GAP)	-1.285
YNL053W	Msg5	Dual-specificity protein phosphatase; regulates/is regulated by Slt2p	-0.704
YOR208W	Ptp2	Nuclear phosphotyrosine-specific phosphatase; inactivate MAPK	0.132
YER075C	Ptp3	Phosphotyrosine-specific protein phosphatase; inactivated MAPK	-1.289
YIL113W	Sdp1	Stress-inducible dual-specificity MAPK phosphatase; negatively regulates Slt2p	-0.214

a : Genes are organized: upstream (top) to downstream (bottom) of the pathway.

b: Log 2 Fold Change taken from Supplementary table S1.

Supplementary table S5: Diploid-specific genes whose expression level is modulated by VPA

	Systematic Name	Standard name[a]	Function	Log2 Fold Change[b]
Up-regulated	YPL250C	ICY2	Protein of unknown function; required for selective and nonselective autophagy	4.189
	YDL039C	PRM7	Pheromone-regulated protein; promoter contains Gcn4p binding elements	3.489
	YGL184C	STR3	Peroxisomal cystathionine beta-lyase	2.882
	YIL056W	VHR1	Transcriptional activator; Involved in response to low biotin concentrations	2.673
	YHR216W	IMD2	Inosine monophosphate dehydrogenase	2.495
	YOL152W	FRE7	Ferric reductase with similarity to Fre2p	2.273
	YJL217W	REE1	Cytoplasmic protein involved in the regulation of enolase (ENO1)	1.908
	YKL071W		Protein of unknown function	1.887
	YEL071W	DLD3	D-lactate dehydrogenase	1.742
	YOL091W	SPO21	Component of the meiotic outer plaque of the spindle pole body	1.462
	YDR523C	SPS1	Protein serine/threonine kinase; expressed at the end of meiosis	1.334
	YOR298W	MUM3	Protein of unknown function involved in outer spore wall organization	1.334
	YPR001W	CIT3	Dual specificity mitochondrial citrate and methylcitrate synthase	1.303
	YOR338W		Protein of unknown function	1.264
	YCR005C	CIT2	Citrate synthase	1.218
	YMR017W	SPO20	Meiosis-specific subunit of the t-SNARE complex	1.199
	YOR255W	OSW1	Protein involved in sporulation	1.194
	YBR072W	HSP26	Small heat shock protein with chaperone activity; suppress unfolded proteins aggregation	1.168
	YBR092C	PHO3	Constitutively expressed acid phosphatase similar to Pho5p; hydrolyzes thiamin phosphates	1.083
	YGL170C	SPO74	Component of the meiotic outer plaque of the spindle pole body	0.999
Down-regulated	YCL014W	BUD3	GEF for Cdc42p. Activated Cdc42p in early G1	-1.001
	YGR269W		Dubious open reading frame; unlikely to encode a functional protein, based on available experimental and comparative sequence data	-1.007
	YLR098C	CHA4	DNA binding transcriptional activator	-1.021
	YAR064W		Protein of unknown function	-1.023
	YIL166C		Putative protein with similarity to allantoate permease (Dal5p) subfamily	-1.024
	YMR118C	SHH3	Putative mitochondrial inner membrane protein of unknown function	-1.036
	YJL019W	MPS3	Nuclear envelope protein	-1.107
	YOL052C-A	DDR2	Multi-stress response protein; DDR2 as a paralog	-1.136
	YJL157C	FAR1	CDK inhibitor and nuclear anchor; Far1p sequesters the GEF Cdc24p in the nucleus; degradation after phosphorylation by Cdc28-Cln; phosphorylation of Far1p by MAPL Fus3p results in association with, and inhibition of Cdc28p-Cln	-1.141

	YLR385C	SWC7	Component of the nuclear Swr1 complex that is involved in chromatin remodeling	-1.178
	YFL040W		member of the sugar porter family; may have a role in intracellular sterol transport	-1.210
	YJL214W	HXT8	Protein of unknown function with similarity to hexose transporters	-1.211
	YGR140W	CBF2	Essential kinetochore protein	-1.225
	YKR045C		Protein of unknown function	-1.268
	YMR078C	CTF18	Subunit of a complex with Ctf8p; subunits with Replication Factor C and is required for sister chromatid cohesion; may have overlapping functions with Rad52p in the DNA damage replication checkpoint	-1.269
	YOL104C	NDJ1	Protein that regulated meiotic SPB cohesion and telomere clustering	-1.290
	YNL270C	ALP1	Arginine transporter	-1.293
	YFL051C		Protein of unknown function	-1.305
	YDL227C	HO	Site-specific endonuclease; required for gene conversion at the MAT locus through the generation of a ds DNA break; expression restricted to mother cells in late G1	-1.343
	YGR014W	MSB2	Mucin family member involved in various signaling pathways; function as osmosensor in the Sho1p-mediated HOG pathway; functions in Cdc42p- and MAP kinase-dependent filamentous growth signaling pathway	-1.390
	YJR053W	BFA1	Component of the GTPase-activating Bfa1p-Bub2p complex; involved in multiple cell cycle checkpoint pathways that control exit from mitosis	-1.404
	YNR070W	PDR18	Putative transporter of the ATP-binding cassette (ABC) family; role in plasma membrane sterol incorporation; implicated in pleiotropic drug resistance	-1.456
	YPL167C	REV3	Catalytic subunit of DNA polymerase zeta; involved in translesion syntheses during post-replication repair; required for mutagenesis induced by DNA damage; involved in double-strand break repair	-1.461
	YMR232W	FUS2	Protein involved in regulating the termination of mating projection growth as well as karyogamy during mating; localizes to nucleus and mating projection tip	-1.601
	YPR054W	SMK1	MAP kinase involved in regulation of sporulation and spore wall assembly; autophosphorylates	-1.635
	YNL279W	PRM1	Pheromone-regulated multispanning membrane protein; involved in membrane fusion during mating; localizes to the shmoo tip; regulated by Ste12p	-1.918
	YDR274C		Dubious open reading frame; unlikely to encode a functional protein, based on available experimental and comparative sequence data	-2.094
	YMR175W	SIP18	Phospholipid-bindinghydrophilin; essential to overcome desiccation-rehydration process; expression is induced by osmotic stress	-2.442

a:Data for diploid-specific genes are from Galitski, T., Saldanha, A. J., Styles, C. A., Lander, E. S. & Fink, G. R. Ploidy Regulation of Gene Expression. *Science* **285,** 251–254 (1999). Up- and down-regulated genes (diploid-specific and VPA-induced) were compared in a Venn diagram (see Supplementary Figure S5); genes that are in both datasets are presented here.

b: Log 2 Fold Change is data from supplementary table S1 and represent VPA-induced change in expression.
